# TFmotifView: a webserver for the visualization of transcription factor motifs in genomic regions

**DOI:** 10.1093/nar/gkaa252

**Published:** 2020-04-23

**Authors:** Clémentine Leporcq, Yannick Spill, Delphine Balaramane, Christophe Toussaint, Michaël Weber, Anaïs Flore Bardet

**Affiliations:** CNRS, University of Strasbourg, UMR7242 Biotechnology and Cell Signaling, Illkirch 67412, France; CNRS, University of Strasbourg, UMR7242 Biotechnology and Cell Signaling, Illkirch 67412, France; CNRS, University of Strasbourg, UMR7242 Biotechnology and Cell Signaling, Illkirch 67412, France; CNRS, University of Strasbourg, UMR7242 Biotechnology and Cell Signaling, Illkirch 67412, France; CNRS, University of Strasbourg, UMR7242 Biotechnology and Cell Signaling, Illkirch 67412, France; CNRS, University of Strasbourg, UMR7242 Biotechnology and Cell Signaling, Illkirch 67412, France

## Abstract

Transcription factors (TFs) regulate the expression of gene expression. The binding specificities of many TFs have been deciphered and summarized as position-weight matrices, also called TF motifs. Despite the availability of hundreds of known TF motifs in databases, it remains non-trivial to quickly query and visualize the enrichment of known TF motifs in genomic regions of interest. Towards this goal, we developed TFmotifView, a web server that allows to study the distribution of known TF motifs in genomic regions. Based on input genomic regions and selected TF motifs, TFmotifView performs an overlap of the genomic regions with TF motif occurrences identified using a dynamic *P*-value threshold. TFmotifView generates three different outputs: (i) an enrichment table and scatterplot calculating the significance of TF motif occurrences in genomic regions compared to control regions, (ii) a genomic view of the organisation of TF motifs in each genomic region and (iii) a metaplot summarizing the position of TF motifs relative to the center of the regions. TFmotifView will contribute to the integration of TF motif information with a wide range of genomic datasets towards the goal to better understand the regulation of gene expression by transcription factors. TFmotifView is freely available at http://bardet.u-strasbg.fr/tfmotifview/.

## INTRODUCTION

The regulation of gene expression is mediated by the binding of transcription factors (TFs) to regulatory regions (1). They contain a DNA binding domain, which recognizes short specific DNA sequence motifs (6–12 bp). Hundreds of TF binding motifs have been determined experimentally using *in vitro* assays such as systematic evolution of ligands by exponential enrichment (SELEX) (2) or high-throughput SELEX (3) and protein-binding microarrays (PBM) (4,5), as well as *in vivo* approaches such as chromatin immunoprecipitation followed by high throughput sequencing (ChIP-seq) (6). They have been summarized as position-weight matrices (PWMs) (7) and are freely available in TF motif databases such as JASPAR (8), *cis*-BP (4) and HOCOMOCO (9).

In order to identify TF motifs in genomic regions of interest, several approaches are available. A *de novo* motif search allows to identify TF motifs enriched directly from the input sequences without prior knowledge. For example, it can be applied to derive motif PWMs from TF ChIP-seq data. Several methods have been developed for *de novo* motif search such as MEME-ChIP from the MEME Suite (10), peak-motifs from RSAT (11) or HOMER (12). Once a motif is identified, it can be compared to known TF motifs found in databases, which is often directly performed by the *de novo* motif search methods.

As an alternative, a known motif search can be performed by taking advantage of the hundreds of motif PWMs from public databases to identify TF motifs enriched in genomic regions of interest. Several methods have been developed for known motif search such as AME from the MEME Suite (13), i-*cis*Target (14), Pscan-ChIP (15) or HOMER (12). The use of known motifs, allows to perform further analyses using the genomic coordinates of the TF motif occurrences such as the identification of motifs at specific positions within the genomic regions. Such analyses can for example be performed by CENTRIMO from the MEME Suite (16).

Despite the large amount of TF motifs available in public databases, their integration with genomic regions of interest remains non-trivial for researchers with no coding experience. Furthermore, the availability of online tools, which take advantage of such valuable data, remains sparse and often lacks graphical representation of the results. Here, we present TFmotifView, a web server that allows to study the distribution of known TF motifs in genomic regions. Using the genomic coordinates of the regions of interest and selected TF motifs, TFmotifView performs an overlap of the regions with TF motif occurrences already pre-processed on our servers. It then generates three different outputs: (i) an enrichment table and scatterplot calculating the significance of TF motif occurrences in genomic regions compared to control regions, (ii) a genomic view of the organisation of TF motifs in each genomic region and (iii) a metaplot summarizing the position of TF motifs relative to the center of the regions. The application is designed for the analysis of TF ChIP-seq peak regions and can be applied to histone ChIP-seq peaks, DNase-seq or ATAC-seq open chromatin regions, gene promoters or any other set of genomic regions of interest.

## MATERIALS AND METHODS

### Transcription factor motif occurrences

TFmotifView relies on known TF motifs from the curated, non-redundant, vertebrates JASPAR CORE 2020 database (8). JASPAR position-weight matrices were used to scan each reference genome (human hg38, hg19 and mouse mm10, mm9) for motif occurrences using MAST from the MEME Suite (17). Since each motif has a different complexity, we used the motif information content (18) to derive a dynamic *P*-value threshold for calling motif occurrences (Supplementary Figure S1A–C). Each motif information content IC is defined using the frequency *f_i,b_* at each position *i* for each base *b* assuming an equal base frequency in the whole genome *p_b_* of 0.25:

$$\begin{equation*}IC\ = \mathop \sum \limits_i \mathop \sum \limits_b {f_{i,b}} \times {\log _2}\frac{{{f_{i,b}}}}{{{p_b}}}\ \end{equation*}$$

The *P*-value threshold is then derived following:

$$\begin{equation*}p{\hbox{-}}{\rm value}\ = \ \frac{1}{{{2^{{\rm IC}}}}}\end{equation*}$$

The *P*-value thresholds derived from the motif's information content were tested using ChIP-seq datasets of TFs expressed in mouse embryonic stem cells (see Supplementary Figure S1). We used the following publicly available datasets: KLF4 (ChIP-Atlas ID ERX1965624), MYC (GEO ID GSM1171649), GABPA (GEO ID GSM3258761), NRF1 (GEO ID GSM1891641), OCT4 (ChIP-Atlas ID ERX1965633), SOX2 (GEO ID GSM288347), SP3 (GEO ID GSM3258757), ESRRB (GEO ID GSM288355), CTCF (GEO ID GSM747534), SP1 (GEO ID GSM3258754) and REST (GEO ID GSM671094) and processed them following the same pipeline as our example data (see below).

We further clustered highly similar motifs using TOMTOM from the MEME Suite (19) and the *hclust* function in R. This led to 747 motifs distributed in 180 clusters (see supplementary data). Additionally, specific motifs can be provided as input using their exact genomic sequence (limited to 6–12 bp).

### Example data

As input genomic regions, we used NRF1 ChIP-seq peaks in mouse embryonic stem cells from our previous study (20) available on GEO (ID GSM1891641). The data was aligned to the mouse reference genome assembly mm10 using bowtie2 (21) and peaks were called using peakzilla (22). Peaks are centered on their summits by peakzilla by default. Selected motifs from the JASPAR database are NRF1 (MA0506.1), the TF of interest, as well as other TFs expressed in mouse embryonic stem cells: MYCN (MA0104.4), GABPA (MA0062.3), CTCF (MA0139.1), REST (MA0138.2) and SP1 (MA0079.3). Control regions are generated randomly (see below).

### Control regions

Global control regions are selected randomly within regions with matched G+C content (using windows of 500 bp sliding by 100 bp containing 0–19, 20–29, 30–34, 35–39, 40–44, 45–49, 50–54, 55–59, 60–69, 70–79 or 80–100 G+Cs per 100 bp), uniquely mappable regions (from 50 bp reads) and not blacklisted regions (23) using the tool *shuffleBed* from BEDTools (24). Local control regions are generated as the regions immediately flanking the genomic regions of interest (half the region size on each side). The significance of enrichment of motifs in genomic regions compared to control regions is given by the hypergeometric *P*-value using *phyper* in R.

### Data analyses

The overlap between input genomic and control regions and TF motif occurrences is performed using intersectBed from BEDTools (24). The position of gene transcription start sites was derived for all coding gene transcripts from the ENSEMBL genome annotation (Homo_sapiens.GRCh38.87 for hg38, Homo_sapiens.GRCh37.71 for hg19, Mus_musculus.GRCm38.87 for mm10 and Mus_musculus.NCBIM37.67 for mm9). Genomic tracks for the UCSC genome browser (25) are generated using bedToBigBed (26). Further data processing, statistics and plots are generated using R (https://www.R-project.org/). Interactive plots are generated using plot_ly in R (https://plot.ly/).

### Web server implementation

TFmotifView was implemented in R using the shiny package (https://shiny.rstudio.com/) and the following libraries: shinydashboard, RColorBrewer, stringr, shinyjs, colourpicker, png, DT, data.table, foreach, doMC, shinyBS, htmltools and plotly. TFmotifView was deployed using the open-source Shiny Server and runs on a dedicated server using an Intel Xeon Silver CPU (10 cores, 20 threads, 2.2 GHz and 96GB RAM). In order to enable simultaneous connections, TFmotifView was containerized using Docker (https://www.docker.com/; image deposited on DockerHub) and using Traefik as load-balancer (https://docs.traefik.io/).

### Performance

Calculations for the example data (7167 genomic regions of 218bp, 6 TF motifs) take less than a minute to run. Only one instance of TFmotifView can be run per user. A URL is provided to come back to the results at a later point once the calculations have finished.

## RESULTS

### Input data and settings

**Figure 1.**
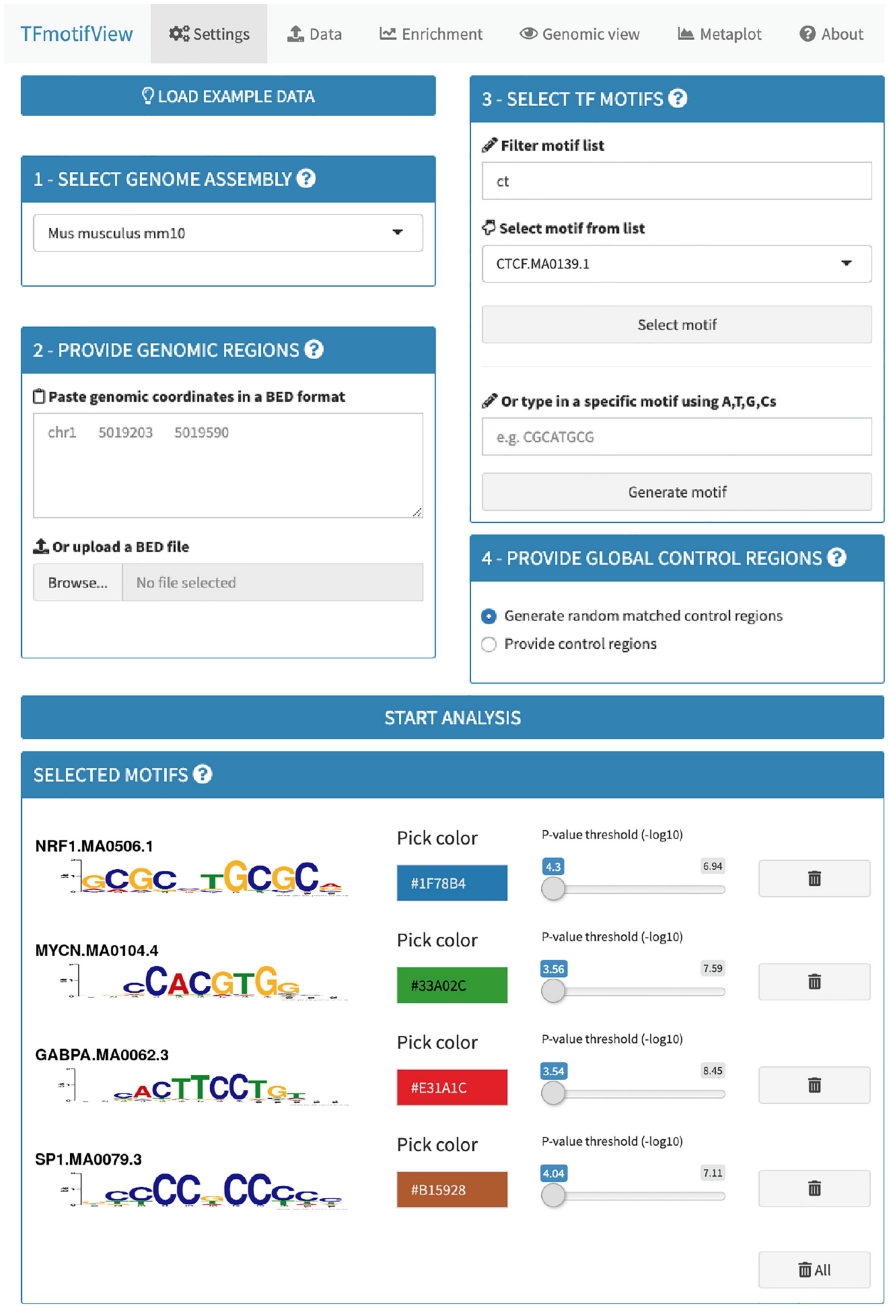
Input and settings. TFmotifView requires two mandatory inputs (1) genome assembly and (2) genomic regions and two optional inputs (3) TF motifs and (4) control regions to run the analysis.

TFmotifView can analyse genomic regions acquired from different sources such as TF ChIP-seq peaks centered on their peak summits (example shown below), regions with no define summit such as histone ChIP-seq regions, DNase-seq or ATAC-seq open chromatin regions, gene promoters or any other set of genomic regions of interest. The different analyses are designed to be run with a minimum of 100 regions and limited to 100 000. However, the generation of the genomic view is optimized when using only one or few regions. The genomic view is optimal for regions up to 1,000 bp although the region size is not limited. TFmotifView requires a minimum of two inputs to run the analysis and two additional inputs are optional (Figure 1).

#### Genome assembly

The first input for TFmotifView is the genome assembly and should match the genomic coordinates of the regions used to search for TF motifs. It is available for the human (hg38, hg19) and mouse (mm10, mm9) genomes.

#### Genomic regions

The second input for TFmotifView is the genomic coordinates of the regions used to search for TF motifs. It should be in a minimal BED format including for each region on a separate line: chromosome, region start and region end, separated by tabulations (additional columns are accepted but not processed).

#### Transcription factor motifs

The third (optional) input for TFmotifView is the motifs that will be used to scan the input genomic regions. They can be selected from the list of available JASPAR motifs, based on their TF names, that were already processed on our servers. Alternatively, exact motif sequences can be provided (between 6 and 12 bp) and will be used to search the genome. Once selected, motif attributes, such as the *P*-value threshold used to scan the genomic regions (default based on its information content) or the color used for visualization, can be updated. In case no motifs are selected, the analysis will run using one motif representative of each motif cluster (see supplementary data).

#### Control regions

The fourth (optional) input for TFmotifView is the genomic coordinates of the regions to be used to contrast the number of input genomic regions containing motif occurrences. Control regions can be either provided as genomic coordinates in a minimal BED format or generated randomly (with matched G+C content, see methods). Strong control regions should have similar genomic characteristics as the genomic regions of interest used as input.

### Analysis of input data

#### Genome G+C and CpG content

Due to the high number of motif occurrences found in genomes (Supplementary Figure S1B), the presence of motifs in the genomic regions of interest could just be due to chance. Therefore, we contrast the results with the number of motifs found in control regions to derive an enrichment as fold change and a significance as *P*-value. We recommend using control regions with similar genomic characteristics as the genomic regions of interest used as input. For example, controls for TF ChIP-seq peaks specific to one condition could be peaks shared in both conditions, controls for promoter regions of genes that are differentially expressed in a specific condition could be promoter regions of genes that remain expressed in both conditions. The genomic feature most likely to influence the results of the motif search is the region G+C content. For example, CpG content is uneven in vertebrate genomes due to the high deamination rate of methylated CpGs (27). Motifs rich in G+Cs are more likely to be found in regions such as CpG islands whereas motifs rich in A+Ts are more likely to be depleted. Therefore, when global control regions are not provided as input, random control regions with matched G+C content will be generated (see methods). Additionally, regions immediately flanking the input regions are also used as local control regions. This local control should retain the characteristics of the input genomic regions unless they mark a break in genomic feature (e.g. flanks of CpG islands will not have a similar CpG content).

#### Analysis of regions G+C content

Genomic regions provided as input as well as generated global control regions are available for download in BED format. An analysis of the G+C content of the regions is performed and summarized as histograms for input and control regions as well as for all bins in the corresponding genome for comparison (see methods). The plots can be downloaded as a PDF file. In case of control regions provided by the user, if the median G+C content of the input regions differs from the control regions by more than five G+Cs in 100 bp, a message warns that the motif enrichment analysis might be biased and generated random control regions with matched G+C content might be a more suitable solution.

### Enrichment of transcription factor motifs in genomic regions over control regions

#### Motif information content

TFmotifView first calculates, for each TF motif, the number of genomic regions containing at least one motif. Motif occurrences are found all over the genome and their number depends on their information content (Supplementary Figure S1A, B). Therefore, rather than using the same lenient *P*-value threshold for all motifs, we use each motif information content (18) to derive a dynamic *P*-value threshold for calling motif occurrences by default (see Materials and Methods). Custom thresholds can be selected on the settings page.

#### Enrichment of transcription factor motifs

**Figure 2.**
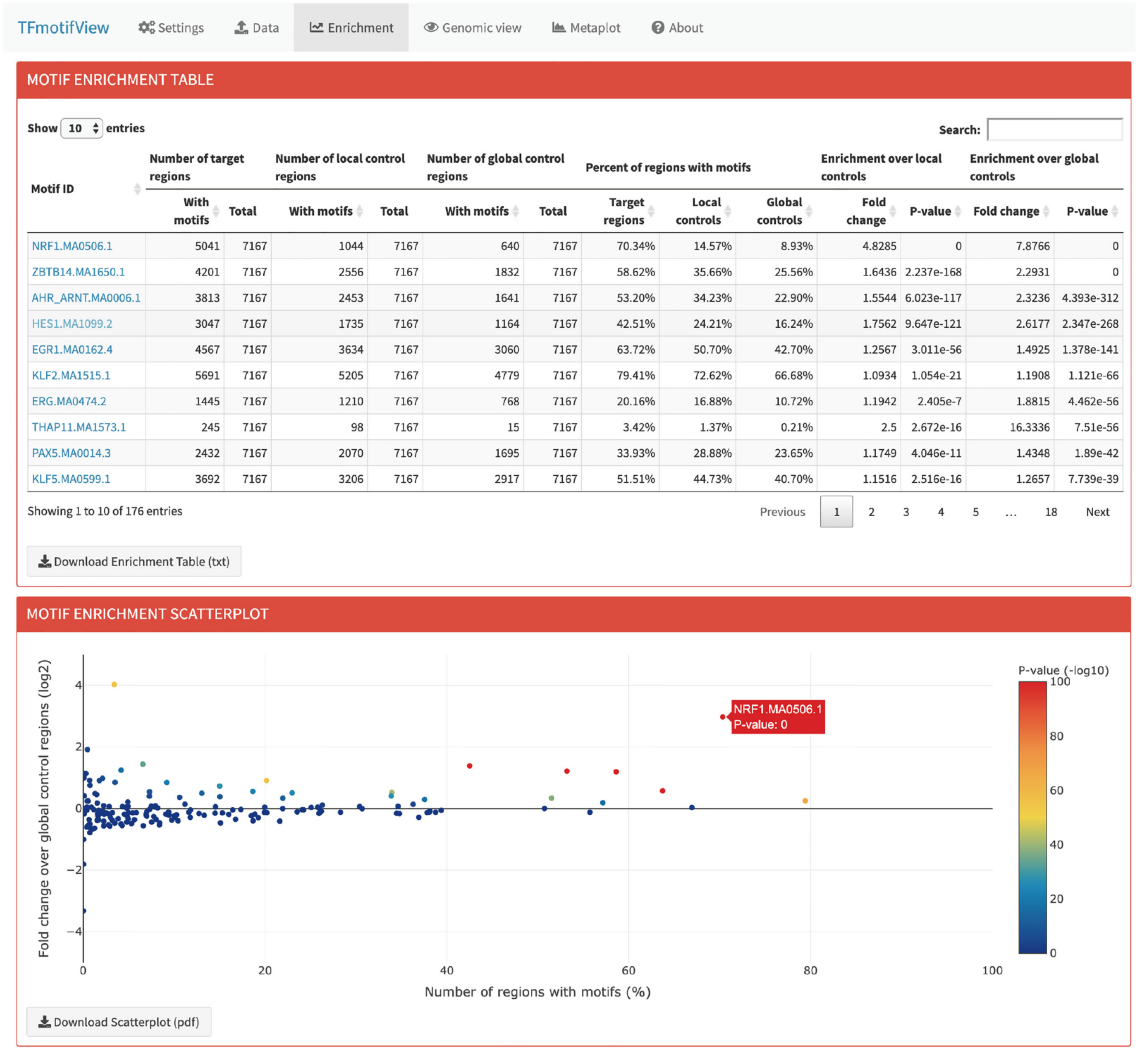
Enrichment of transcription factor motifs in genomic regions over control regions. The number of genomic regions of interest containing each selected motif is compared to the number of local or global control regions using a fold change and hypergeometric *P*-value and represented in a scatterplot.

The motif enrichment table shows for each motif (lines), the number and percent of input and global and local control regions that contain at least one motif (Figure 2). The enrichment of motifs in genomic regions over global or local control regions is then calculated as a fold change with an associated hypergeometric *P*-value. Motifs are ranked according to the global *P*-value but can be ranked according to the column of interest using the arrows. Motif names link to their JASPAR motif page. The table can be downloaded as a tab delimited text file.

The motif enrichment scatterplot represents for each motif (points), the number of input genomic regions containing at least one motif (in percent) compared to their fold change over the global control regions (in log_2_ scale) (Figure 2). The global *P*-value is used to color the points. The plot is interactive and information about the corresponding motif and *P*-value can be obtained by pointing to it with the cursor. The plot can also be downloaded as a PDF file.

#### Results interpretation

As expected, the NRF1 motif is found to be highly enriched in the NRF1 ChIP-seq peaks (Figure 2). About 70% of the peaks have the motif, which is what is usually expected in ChIP-seq experiments since crosslinking artefacts can lead to unspecific peaks without motif. The NRF1 motif enrichment stands out on the scatterplot compare to other TF motifs (*P*-value 0; fold change 4.8). Motifs should be ranked according to their *P*-value. Motifs with high fold changes can occasionally have high *P*-values because they were found in only few input genomic regions (e.g. ZNF143: fold change 2.2, *P*-value 0.1, only 0.15% peaks with motif). Conversely, motifs present in many input and control genomic regions can have a low *P*-value even though their fold change is small (e.g. KLF2: fold change 1.2, *P*-value 1.1 × 10^−66^, 79% of peaks with motif vs. 67% of control peaks with motif).

### Genomic view of transcription factor motifs in genomic regions

#### Motif positions in genomic regions

**Figure 3.**
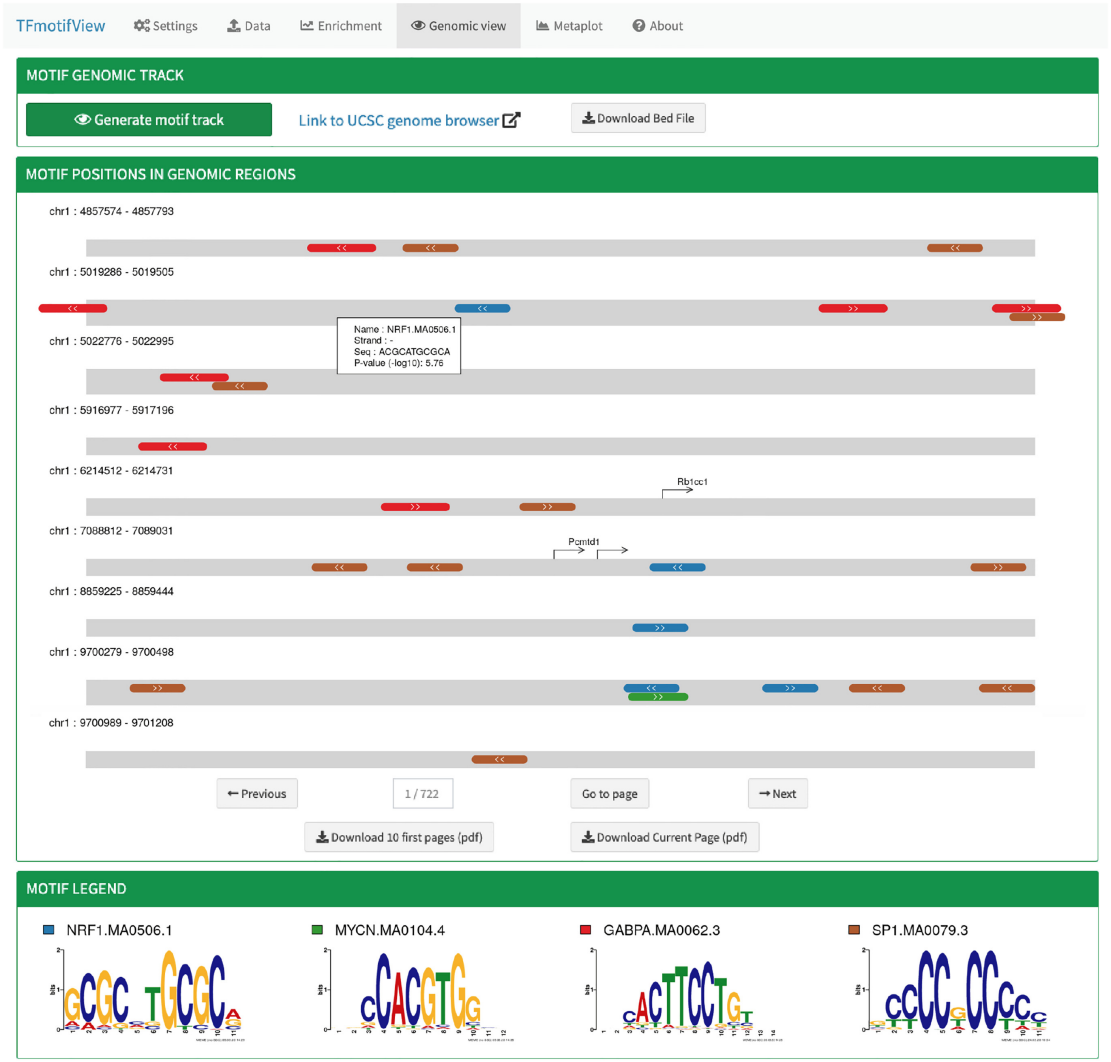
Genomic view of transcription factor motifs in genomic regions. The position and organization of TF motifs is represented as colorful boxes within the genomic regions (gray bars) or can be integrated in the UCSC genome browser.

TFmotifView allows to visualize the position and organisation of the selected motifs within the genomic regions of interest (Figure 3). This visualization is best for analyses using few selected motifs. It enables to inspect the number of motif occurrences, orientation, distance to each other or to gene transcriptional start sites (TSS), exact sequence and *P*-value score. However, it is important to keep in mind that TF motif occurrences are present in large numbers over the genome (Supplementary Figure S1B) and individual motif occurrences will not all represent bona fide TF binding sites. Only regions that contain at least one motif occurrence are displayed. Genomic regions are represented as gray bars scaled according to the maximum region length and specific motifs as colorful boxes. Information about individual motifs such as motif name, strand, exact sequence and *P*-value score, can be accessed by clicking on the motif. Gene transcription start sites of protein coding transcripts are indicated as directional arrows. The legend indicates the motif sequence logos and the colors used for display. Results can be downloaded in a PDF format (limited to maximum 10 pages).

#### Motif genomic track

In order to integrate the motif occurrences with other genomic datasets, a motif track, representing the position of the selected motifs with the selected *P*-value threshold along the whole genome, can be generated and directly uploaded onto the UCSC genome browser or downloaded as a BED file.

#### Results interpretation

The NRF1 ChIP-seq peak regions contain many occurrences of the NRF1 and SP1 motifs (70% and 51% respectively) and fewer GABPA and MYCN motifs (30% and 15% respectively) (Figure 3). On the genomic view, we observe that the NRF1 motifs are located in the center of the peak regions whereas the SP1 peaks are distributed over the whole regions. Mainly one NRF1 motif occurrence is observed in each genomic region whereas several occurrences of the SP1 motif are often observed. This matches the higher enrichment of SP1 motifs in control regions (38% of global control regions have the SP1 motif versus 9% for NRF1). Overall, we do not observe in that case any specific arrangement of the different motifs relative to the region, genes TSS or other motif occurrences. We observe a case of overlap of the NRF1 and MYCN motifs although their motifs logos are quite distinct.

### Enrichment of transcription factor motifs relative to the center of genomic regions

#### Metaplot of motif enrichment

**Figure 4.**
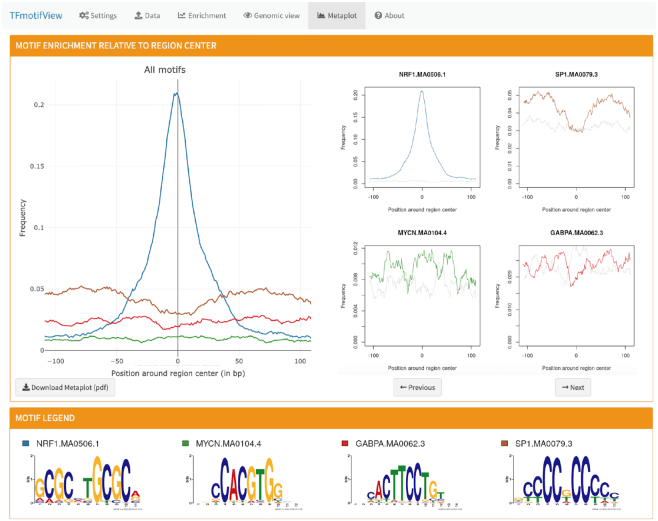
Metaplot of transcription factor motif enrichment relative to genomic regions centers.

TFmotifView allows to visualize the enrichment of the selected motifs relative to the center of the regions as a metaplot (Figure 4). This representation is relevant for genomic regions that have a defined center such as ChIP-seq peak regions where the center/summit of the peaks is expected to correspond to the position where the TF binds to its motif, or regions which are centered on gene transcription start sites. For each motif, the percent of regions having a motif is calculated at each position along the centered genomic regions. The left panel compares the enrichment for all selected motifs in the genomic regions of interest (colorful lines) whereas the right panel shows the enrichment of each motif separately compared to the control regions (gray line). The legend indicates the motif sequence logos and the colors used for display. Results can be downloaded in a PDF format.

#### Results interpretation

As expected, the NRF1 ChIP-seq peak regions are enriched in NRF1 motifs over control regions specifically at the peak summits (Figure 4). SP1 motifs are enriched over control regions across the whole regions except at the peaks summits where NRF1 is enriched. This overall enrichment of SP1 motifs is explained by the enrichment of NRF1 binding sites in regions with high G+C content such as CpG island promoters, where SP1 motifs are enriched. In contrast, the GABPA and MYCN motifs are only moderately enriched across the whole NRF1 peak regions.

## DISCUSSION

Understanding the regulation of gene expression by TFs is an active field of research. TF motifs are a great resource to better comprehend the binding specificities of TFs. However, they should not only be exploited in a static genome but rather integrated to a wide range of condition-specific genomic datasets. Toward this aim, we developed TFmotifView, a web server that allows to study the distribution of known TF motifs in genomic regions. As a webserver, the analyses from TFmotifView are accessible to researchers with no coding experience or compute resources. It directly takes genomic coordinates as input, which are generally provided by common genomic data processing pipelines. It uses an original approach to set the default *P*-value threshold dynamically based on each motif information content or allows the user to set the threshold of their choice. It allows to integrate the corresponding motif track (with the selected *P*-value threshold) into the UCSC genome browser for integration with other genomic datasets. Finally, it generates graphical representations of the motif enrichments, which allows for a better interpretation of the results. These different features represent an added value compared to other existing methods available to study TF motif distributions in genomic regions (Table 1). We believe that TFmotifView will contribute to integrate TF motif information with a wide range of genomic datasets and is relevant for a broad range of questions toward the goal to better understand the regulation of gene expression by transcription factors.

**Table 1.** Comparison of TFmotifView features to other known motif search tools

**Tools**	**TFmotifView**	**AME (MEME suite)**	**CENTRIMO (MEME suite)**	**i-cisTarget**	**Pscan-ChIP**	**HOMER**	**JASPAR database**
**Reference**	This article	13	16	14	15	12	8
**Online web server**	YES	YES	YES	YES	YES	NO (command line)	NA
**Input format**	BED regions	FASTA sequences	FASTA sequences (same length)	BED regions	BED regions (150bp)	BED regions (200bp or all)	NA
**Genome**	hg38, hg19 mm10, mm9	No genome required	No genome required	hg19 mm9 dm3,dm6 daphnia	hg38, hg19, hg18 mm10, mm9	Available locally	hg38, hg19 mm10 dm6, ce10 danRer11 araTha1 sacCer3
**Motif database**	JASPAR or provided	JASPAR, others or provided	JASPAR or others	Several	JASPAR, TRANSFAC or provided	Custom	JASPAR
**Motif score threshold**	Dynamic *P*-value using information content (most below 10^−3^) or custom	hit threshold 0.25	5 bits - likelyhood ratio 32 (score does not affect results)	No threshold (motifs are used to define i-cisTarget regulatory regions)	No threshold (comparing best score in input regions vs. background regions)	*P*-value 0.05	NA
**Uses control regions**	YES (sampled from mappable, not blacklisted regions or provided)	YES (shuffled input FASTA sequences)	NO	NO	YES (sampled from ENCODE DNase regions)	YES (large number sampled from regions within 50Kb of gene TSS or provided)	NA
**Corrects for G+C content**	YES	YES (preserves 2mers)	NO	NO	NO (allows from mixed or promoter regions)	YES (by G+C or CpG)	NA
**Table of motif enrichment**	YES	YES	NO	YES	YES	YES	NA
				(strategy does not allow to provide the number of regions having a motif)			
**Measure of enrichment**	Hyper-geometric *P*-value	Fisher's exact adjusted *P*-value	Binomial e-value	Normalized enrichment score	T-test *P*-value	Binomial *P*-value	NA
**Graphical representation of motif enrichment**	YES (enrichment vs foldchange for all motifs)	NO	NO	YES (AUC per motif)	NO	NO	NA
**Genomic view**	Custom + UCSC genome browser (choice of motif and threshold)	NO	NO	UCSC genome browser for predicted regions	Link to JASPAR track	NO	UCSC genome browser (all motifs at default threshold)
**Enrichment at region center**	YES	NO	YES	NO	YES	Script available	NA

## DATA AVAILABILITY

TFmotifView is freely available at http://bardet.u-strasbg.fr/tfmotifview/

## Supplementary Material

gkaa252_Supplemental_Files
