# Supplementary material for: TFmotifView: a webserver for the visualization of transcription factor motifs in genomic regions
Source: Nucleic Acids Res. 2020 Apr 23;48(W1):W208–17. doi: 10.1093/nar/gkaa252 (PMC7319436; doi:10.1093/nar/gkaa252)
Supplement: gkaa252_Supplemental_Files [file gkaa252_supplemental_files.zip › Supplementary_data_tf_motif_clusters.pdf]

Supplementary data - TF motif clusters

TF motifs from JASPAR 2020 were clustered using TOMTOM from MEME Suite and the hclust function in R. This led to 747 motifs distributed in 180 clusters.

cluster 1

ZNF528.MA1597.1

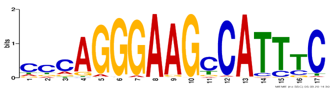

cluster 2

ZNF382.MA1594.1

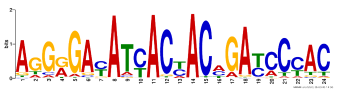

cluster 3

ZBTB32.MA1580.1

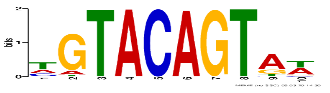

cluster 4

THAP11.MA1573.1

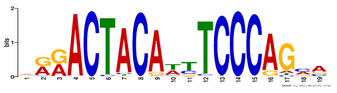

cluster 5

ZKSCAN1.MA1585.1

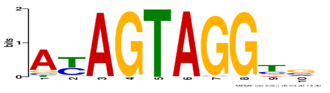

cluster 6

OSR1.MA1542.1

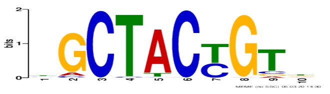

OSR2.MA1646.1

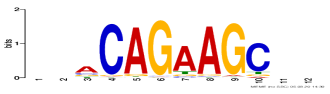

cluster 7

ZNF317.MA1593.1

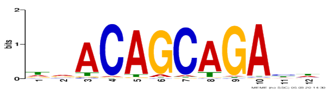

ZIC1_ZIC2.MA1628.1

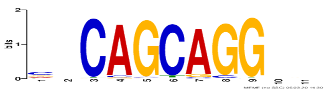

ZIC2.MA1629.1

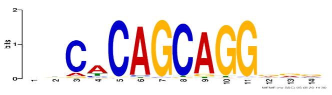

cluster 8

ZSCAN4.MA1155.1

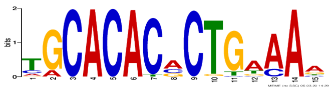

cluster 9

MTF1.MA0863.1

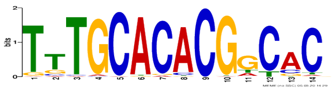

cluster 10

ZSCAN29.MA1602.1

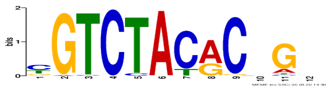

cluster 11

SMAD3.MA0795.1

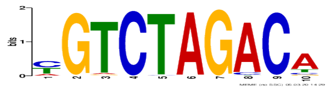

SMAD4.MA1153.1

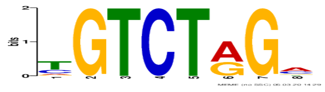

SMAD5.MA1557.1

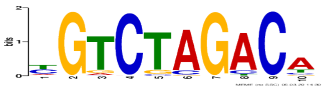

cluster 12

ZNF410.MA0752.1

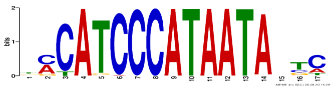

cluster 13

ZNF136.MA1588.1

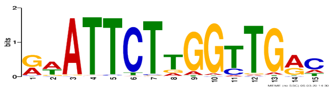

cluster 14

CENPB.MA0637.1

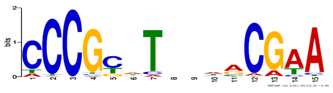

cluster 15

ZFP57.MA1583.1

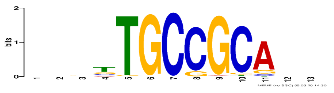

cluster 16

GCM1.MA0646.1

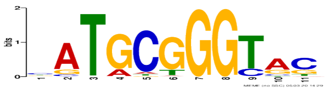

GCM2.MA0767.1

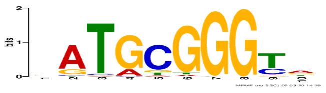

cluster 17

LIN54.MA0619.1

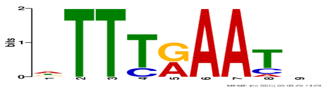

cluster 18

DMRT3.MA0610.1

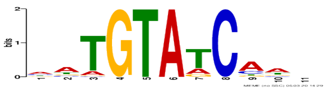

DMRTA2.MA1478.1

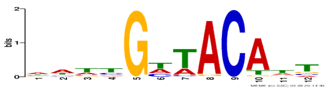

DMRTC2.MA1479.1

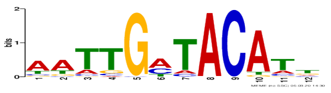

cluster 19

ARID5A.MA0602.1

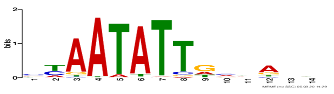

cluster 20

RHOX11.MA0629.1

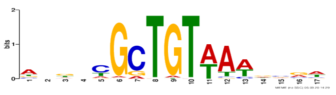

SCRT1.MA0743.2

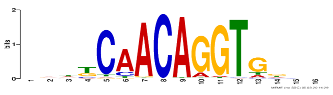

SCRT2.MA0744.2

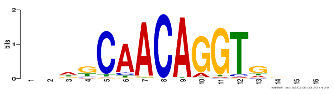

cluster 21

FOXH1.MA0479.1

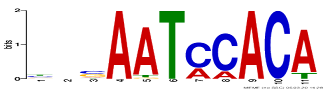

cluster 22

DUX.MA0611.1

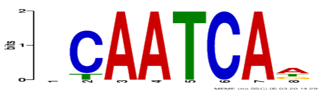

cluster 23

NR2E3.MA0164.1

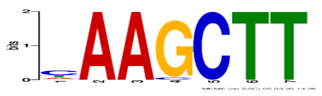

cluster 24

ZNF16.MA1654.1

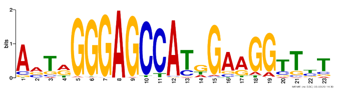

cluster 25

PLAG1.MA0163.1

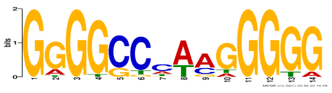

cluster 26

PLAGL2.MA1548.1

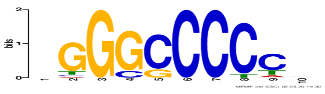

PLAGL1.MA1615.1

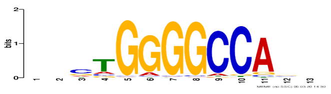

cluster 27

EWSR1_FLI1.MA0149.1

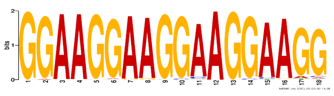

cluster 28

ZNF135.MA1587.1

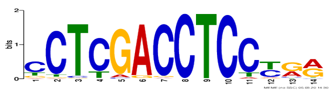

ZNF460.MA1596.1

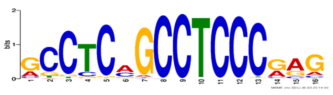

cluster 29

ZNF449.MA1656.1

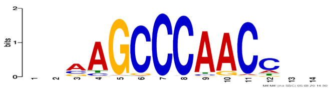

cluster 30

ZFX.MA0146.2

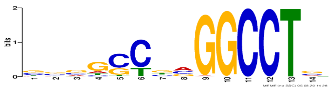

cluster 31

ZNF682.MA1599.1

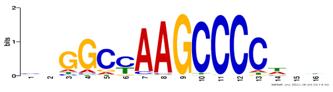

cluster 32

PRDM15.MA1616.1

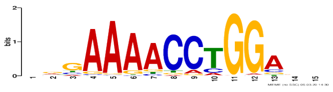

cluster 33

TFCP2.MA0145.3

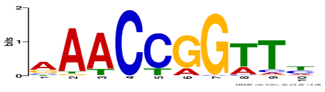

GRHL1.MA0647.1

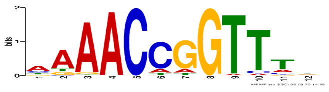

GRHL2.MA1105.2

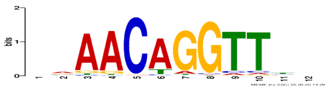

cluster 34

REST.MA0138.2

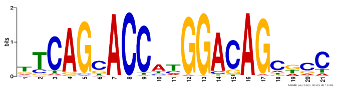

cluster 35

SPZ1.MA0111.1

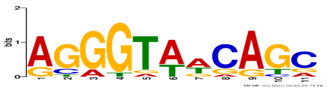

cluster 36

SIX3.MA0631.1

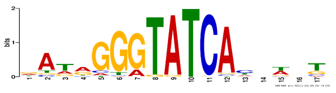

cluster 37

HLTF.MA0109.1

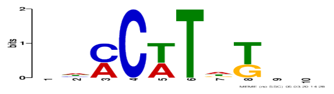

cluster 38

ATOH1.MA0461.2

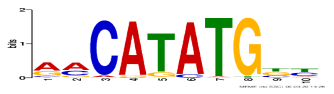

BHLHA15.MA0607.1

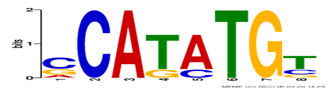

NEUROG1.MA0623.2

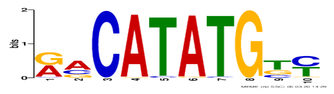

TWIST2.MA0633.1

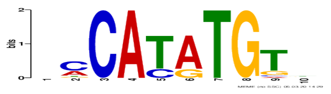

NEUROD2.MA0668.1

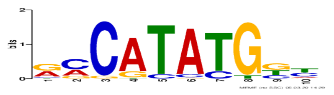

NEUROG2.MA0669.1

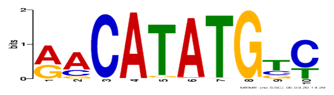

OLIG2.MA0678.1

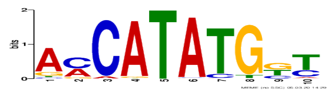

BHLHE23.MA0817.1

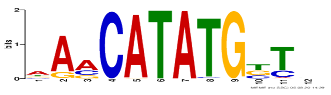

BHLHE22.MA0818.1

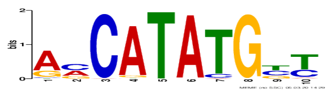

OLIG1.MA0826.1

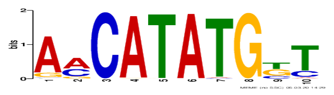

OLIG3.MA0827.1

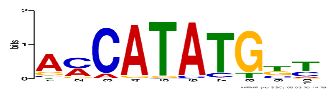

MXI1.MA1108.2

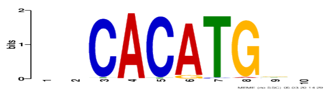

ATOH7.MA1468.1

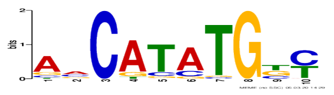

MSGN1.MA1524.1

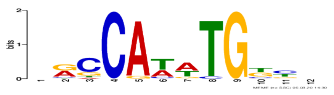

TCF21_2.MA1568.1

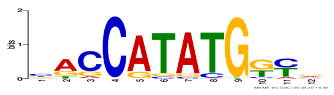

TFAP4_2.MA1570.1

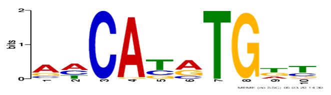

HAND2.MA1638.1

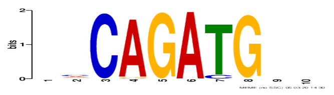

cluster 39

TP53.MA0106.3

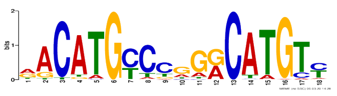

TP63.MA0525.2

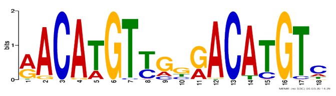

TP73.MA0861.1

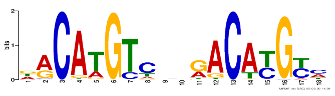

cluster 40

YY1.MA0095.2

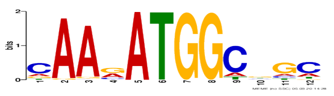

YY2.MA0748.2

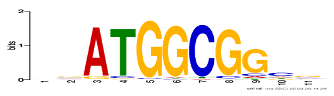

ZFP42.MA1651.1

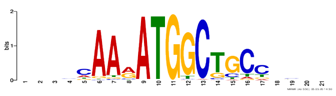

cluster 41

ZNF684.MA1600.1

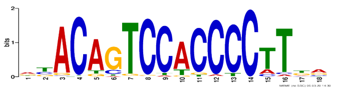

cluster 42

TEAD1.MA0090.3

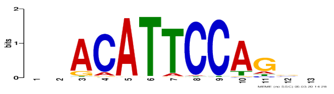

TEAD3.MA0808.1

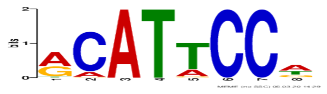

TEAD4.MA0809.2

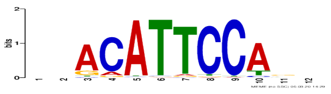

TEAD2.MA1121.1

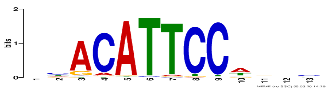

cluster 43

ZNF143.MA0088.2

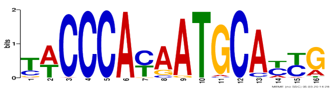

cluster 44

ZNF282.MA1154.1

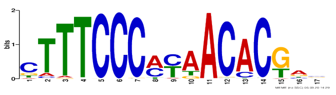

cluster 45

RBPJ.MA1116.1

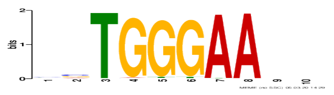

ZNF75D.MA1601.1

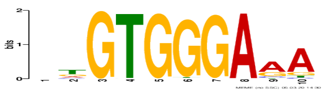

cluster 46

STAT6.MA0520.1

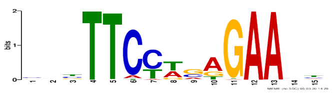

cluster 47

BCL6.MA0463.2

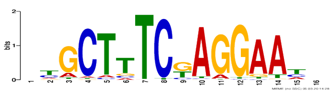

BCL6B.MA0731.1

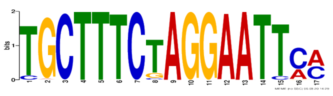

cluster 48

STAT1.MA0137.3

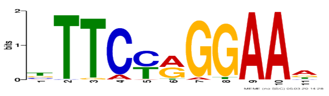

STAT3.MA0144.2

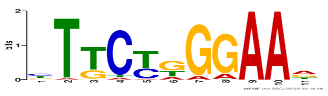

STAT4.MA0518.1

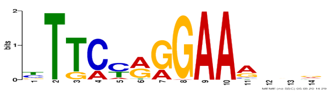

STAT5A_STAT5B.MA0519.1

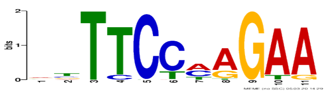

STAT5A.MA1624.1

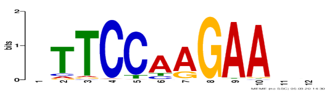

STAT5B.MA1625.1

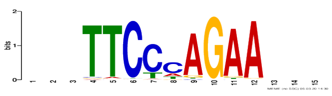

cluster 49

INSM1.MA0155.1

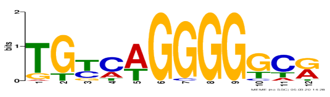

cluster 50

MZF1_2.MA0057.1

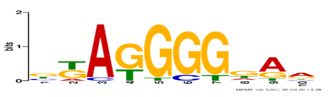

cluster 51

WT1.MA1627.1

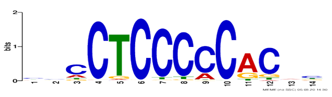

ZNF281.MA1630.1

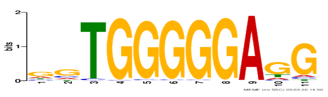

cluster 52

MEF2A.MA0052.4

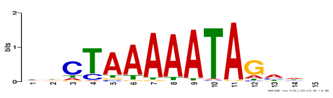

MEF2C.MA0497.1

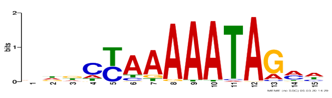

MEF2B.MA0660.1

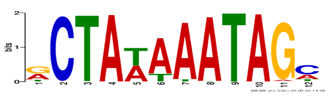

MEF2D.MA0773.1

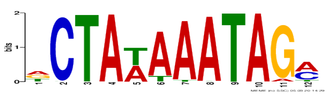

cluster 53

SREBF1.MA0595.1

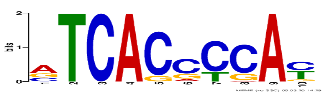

SREBF2.MA0596.1

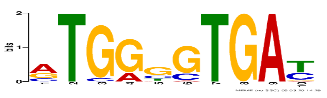

cluster 54

GFI1.MA0038.2

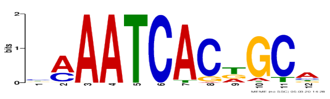

GFI1B.MA0483.1

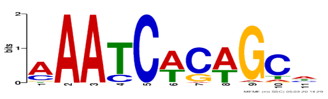

cluster 55

SOX21.MA0866.1

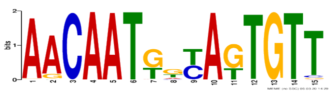

SOX11.MA0869.1

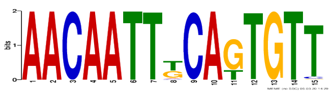

SOX1.MA0870.1

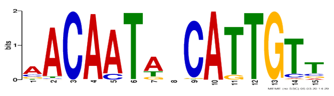

cluster 56

OVOL1.MA1544.1

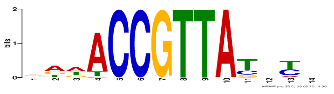

OVOL2.MA1545.1

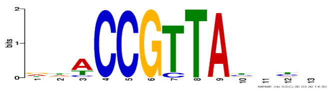

ISL1.MA1608.1

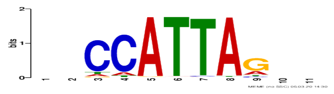

cluster 57

MYBL1.MA0776.1

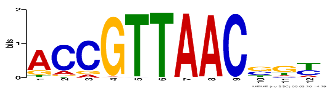

MYBL2.MA0777.1

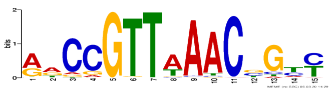

cluster 58

SOX18.MA1563.1

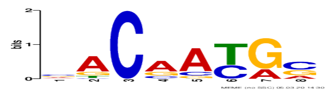

cluster 59

ZNF384.MA1125.1

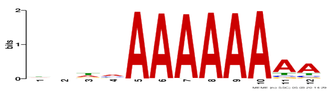

cluster 60

SOX12.MA1561.1

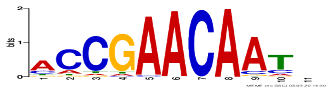

SOX14.MA1562.1

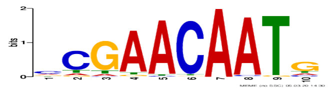

cluster 61

ONECUT1.MA0679.2

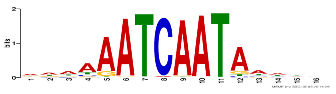

ONECUT2.MA0756.1

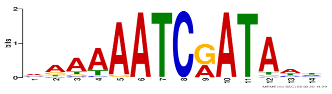

ONECUT3.MA0757.1

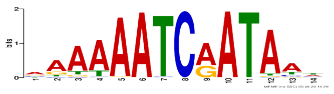

cluster 62

PAX7.MA0680.1

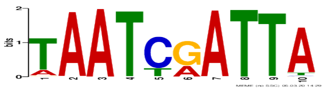

CUX1.MA0754.1

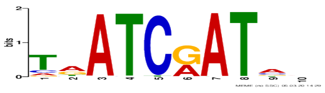

CUX2.MA0755.1

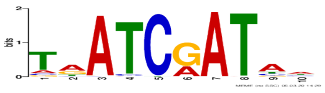

PAX3.MA0780.1

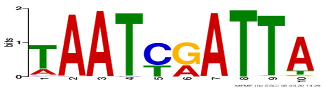

cluster 63

PROX1.MA0794.1

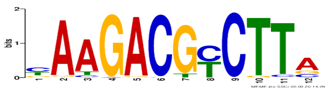

cluster 64

ZBTB7B.MA0694.1

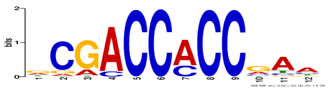

ZBTB7C.MA0695.1

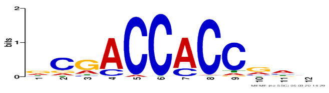

cluster 65

ZIC1.MA0696.1

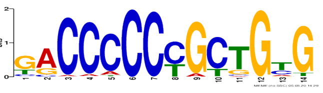

ZIC3.MA0697.1

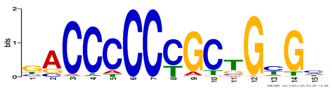

GLI2.MA0734.2

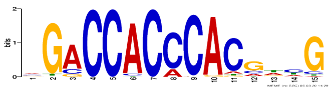

GLIS1.MA0735.1

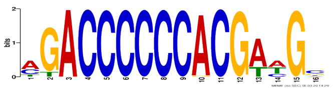

GLIS2.MA0736.1

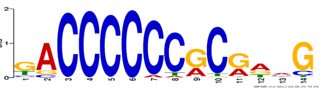

GLIS3.MA0737.1

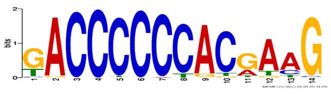

ZIC4.MA0751.1

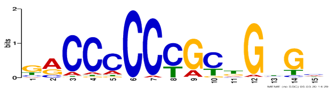

GLI3.MA1491.1

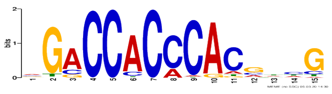

ZIC5.MA1584.1

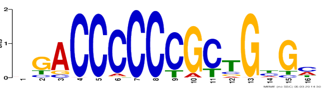

cluster 66

SIX1.MA1118.1

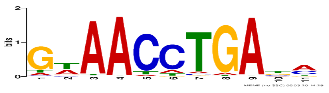

SIX2.MA1119.1

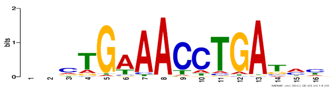

cluster 67

RUNX1.MA0002.2

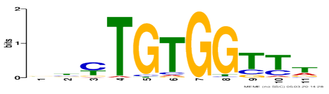

RUNX2.MA0511.2

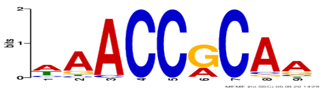

RUNX3.MA0684.2

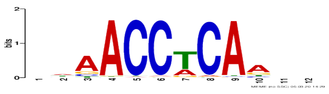

cluster 68

HSF1.MA0486.2

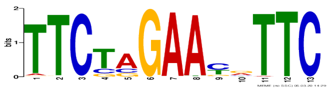

HSF2.MA0770.1

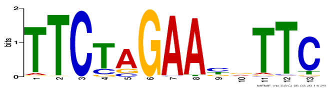

HSF4.MA0771.1

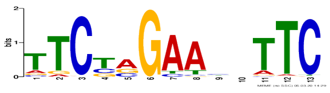

cluster 69

ZBTB6.MA1581.1

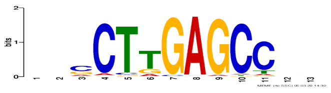

cluster 70

ZBTB12.MA1649.1

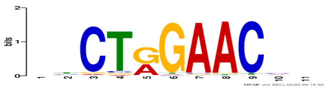

cluster 71

NKX2_3.MA0672.1

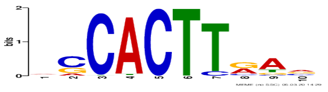

NKX2_8.MA0673.1

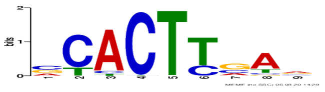

cluster 72

NKX3_2.MA0122.3

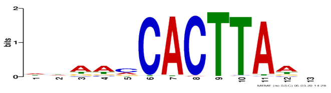

NKX3_1.MA0124.2

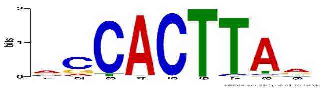

LHX2.MA0700.2

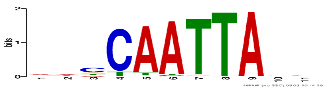

HMX1.MA0896.1

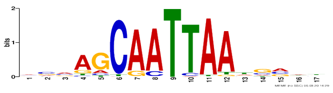

HMX2.MA0897.1

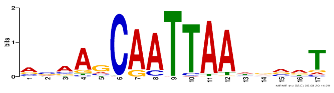

HMX3.MA0898.1

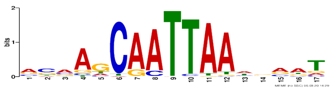

ISL2.MA0914.1

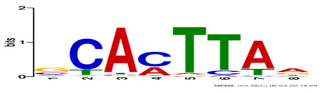

cluster 73

TBP.MA0108.2

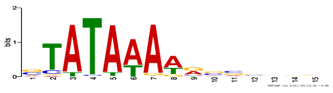

cluster 74

ARID3A.MA0151.1

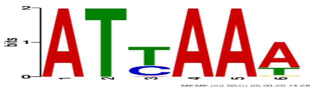

cluster 75

CDX2.MA0465.2

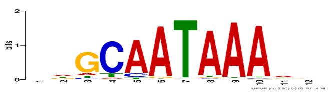

HOXA13.MA0650.2

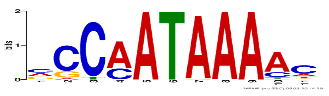

CDX1.MA0878.2

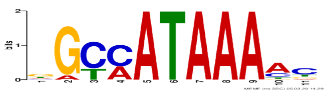

HOXA10.MA0899.1

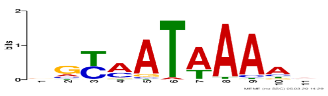

HOXB13.MA0901.2

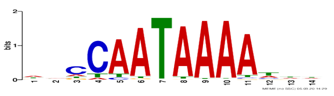

HOXD13.MA0909.2

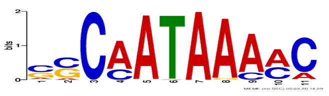

HOXD9.MA0913.2

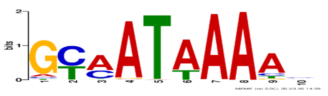

CDX4.MA1473.1

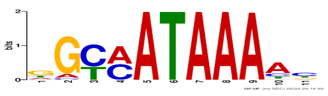

cluster 76

HAND1_TCF3.MA0092.1

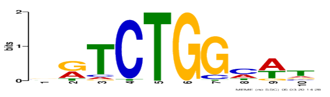

cluster 77

ZBTB26.MA1579.1

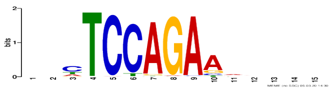

cluster 78

NFIC_TLX1.MA0119.1

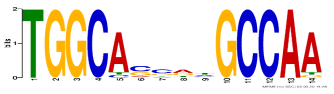

NFIC_2.MA1527.1

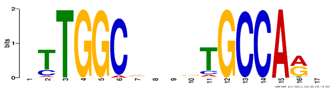

NFIX_2.MA1528.1

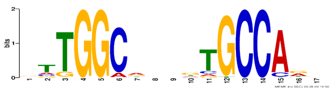

NFIB.MA1643.1

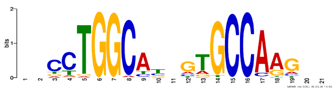

cluster 79

HIC2.MA0738.1

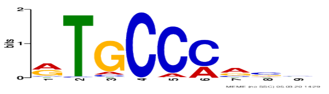

HIC1.MA0739.1

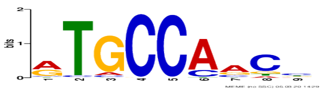

cluster 80

NFIC.MA0161.2

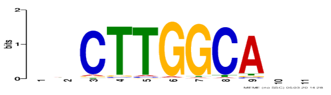

NFIA.MA0670.1

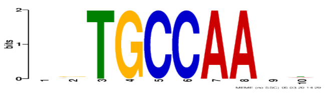

NFIX.MA0671.1

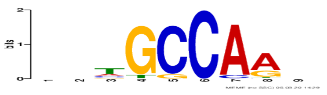

cluster 81

GATA3.MA0037.3

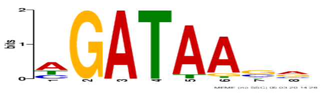

GATA1_TAL1.MA0140.2

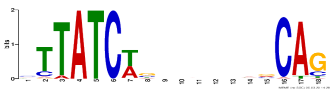

GATA5.MA0766.2

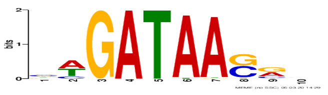

cluster 82

MECOM.MA0029.1

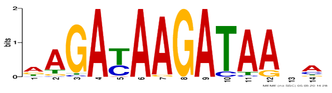

cluster 83

GATA1.MA0035.4

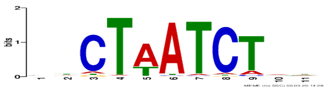

GATA2.MA0036.3

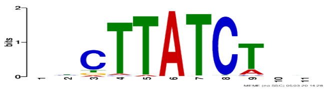

GATA4.MA0482.2

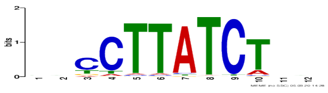

GATA6.MA1104.2

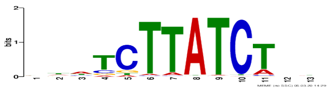

cluster 84

SRF.MA0083.3

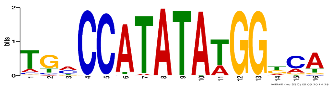

cluster 85

CTCF.MA0139.1

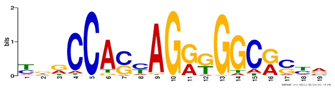

CTCFL.MA1102.2

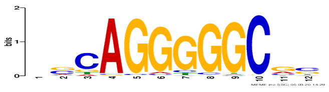

cluster 86

DMRT1.MA1603.1

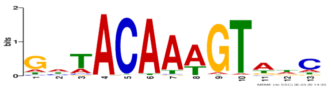

cluster 87

SOX10.MA0442.2

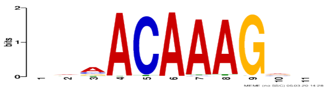

SOX3.MA0514.1

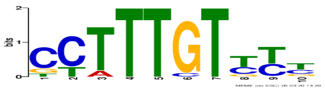

SOX6.MA0515.1

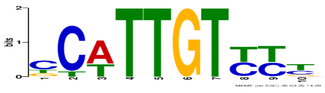

SOX4.MA0867.2

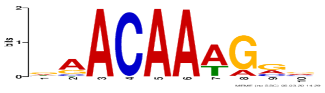

cluster 88

NRF1.MA0506.1

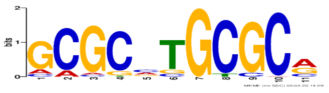

TCFL5.MA0632.2

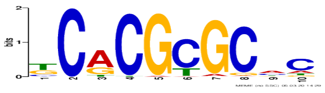

cluster 89

HINFP.MA0131.2

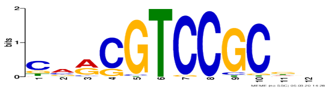

cluster 90

ZBTB14.MA1650.1

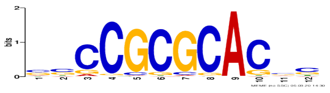

cluster 91

RREB1.MA0073.1

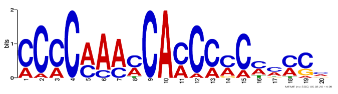

cluster 92

PAX6.MA0069.1

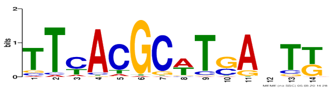

PAX1.MA0779.1

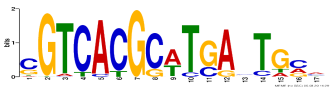

PAX9.MA0781.1

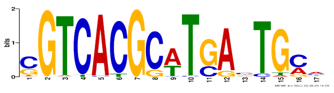

cluster 93

CEBPA.MA0102.4

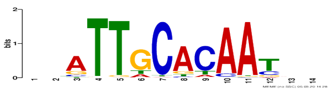

CEBPB.MA0466.2

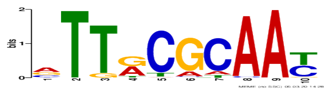

DBP.MA0639.1

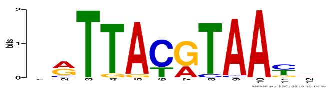

CEBPD.MA0836.2

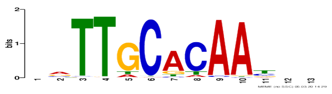

CEBPE.MA0837.1

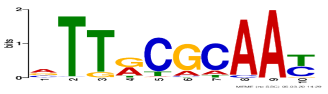

CEBPG.MA0838.1

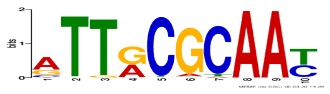

TEF.MA0843.1

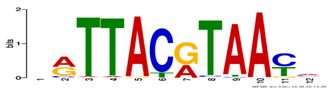

cluster 94

DDIT3_CEBPA.MA0019.1

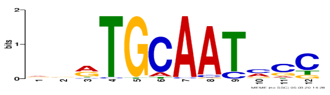

cluster 95

NFIL3.MA0025.2

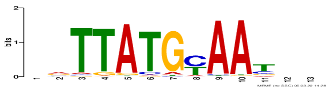

HLF.MA0043.3

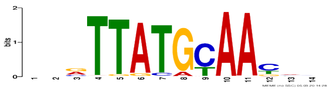

ATF4.MA0833.2

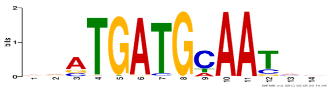

CEBPG_2.MA1636.1

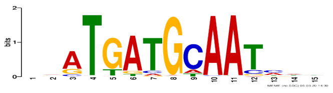

cluster 96

AR.MA0007.3

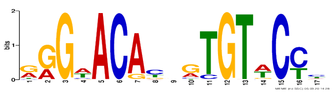

NR3C1.MA0113.3

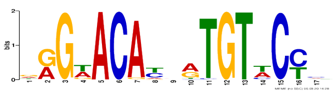

NR3C2.MA0727.1

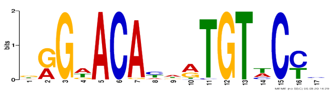

cluster 97

PRDM4.MA1647.1

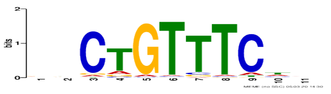

cluster 98

ZNF341.MA1655.1

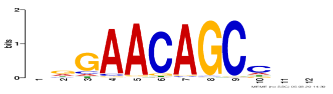

cluster 99

RFX1.MA0509.2

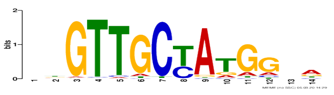

RFX5.MA0510.2

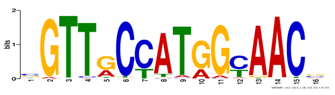

RFX2.MA0600.2

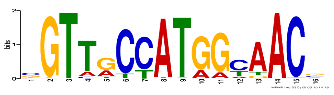

RFX3.MA0798.2

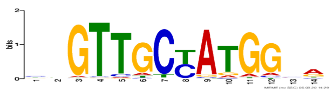

RFX4.MA0799.1

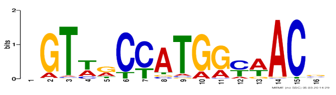

RFX7.MA1554.1

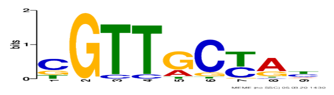

cluster 100

NKX2_5.MA0063.2

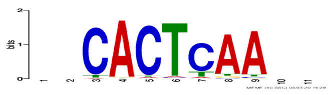

NKX2_5_2.MA0503.1

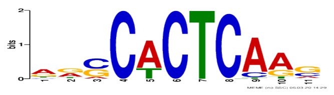

MSANTD3.MA1523.1

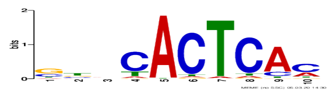

NKX2_2.MA1645.1

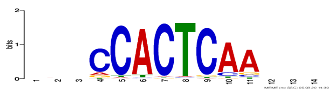

cluster 101

ZNF140.MA1589.1

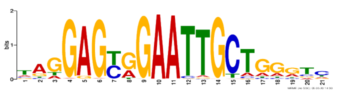

cluster 102

MZF1.MA0056.2

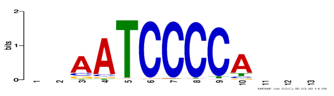

RELB.MA1117.1

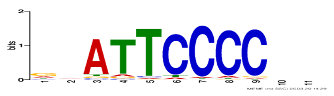

cluster 103

NFATC2.MA0152.1

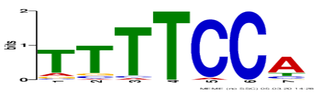

NFAT5.MA0606.1

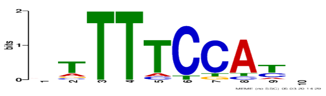

NFATC1.MA0624.1

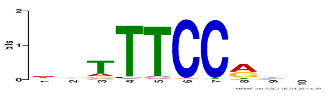

NFATC3.MA0625.1

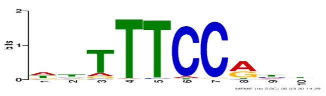

NFATC4.MA1525.1

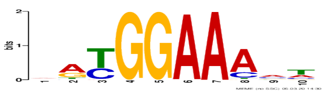

cluster 104

NFKB1.MA0105.4

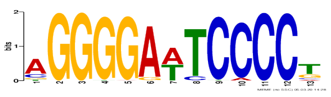

NFKB2.MA0778.1

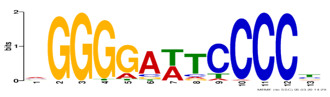

cluster 105

REL.MA0101.1

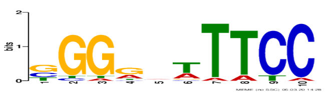

RELA.MA0107.1

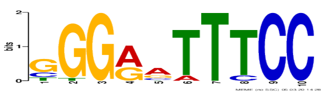

cluster 106

IRF6.MA1509.1

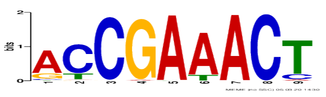

cluster 107

IRF1.MA0050.2

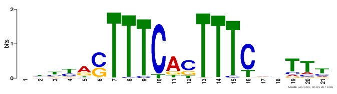

IRF2.MA0051.1

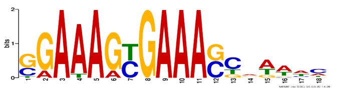

STAT1_STAT2.MA0517.1

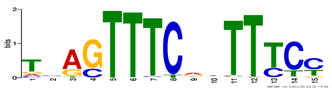

IRF8.MA0652.1

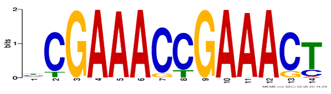

IRF9.MA0653.1

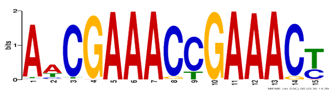

IRF7.MA0772.1

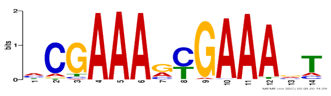

IRF3.MA1418.1

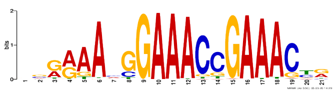

IRF4.MA1419.1

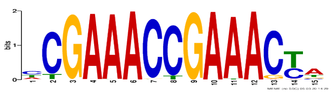

IRF5.MA1420.1

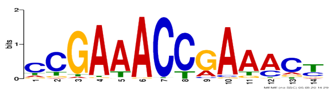

cluster 108

TCF7L2.MA0523.1

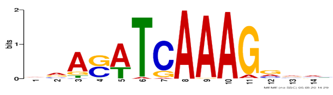

LEF1.MA0768.1

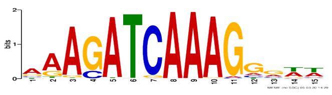

TCF7.MA0769.2

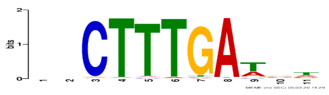

TCF7L1.MA1421.1

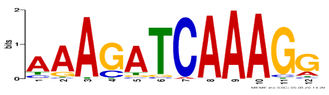

cluster 109

MEIS1.MA0498.2

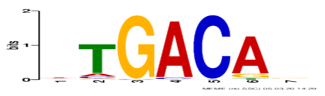

MEIS2.MA0774.1

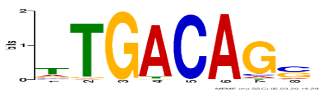

MEIS3.MA0775.1

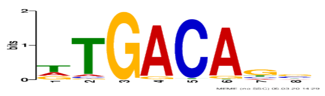

PKNOX2.MA0783.1

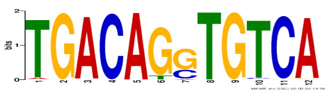

TGIF1.MA0796.1

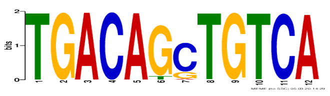

TGIF2.MA0797.1

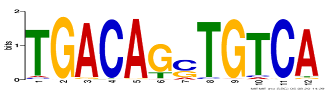

TGIF2LX.MA1571.1

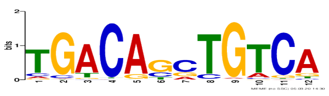

TGIF2LY.MA1572.1

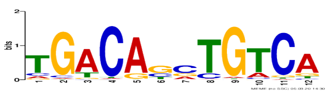

cluster 110

SMAD2_SMAD3_SMAD4.MA0513.1

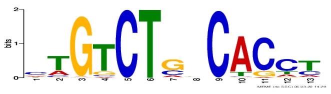

cluster 111

PKNOX1.MA0782.2

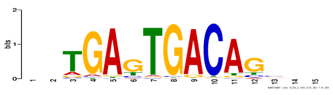

PBX3.MA1114.1

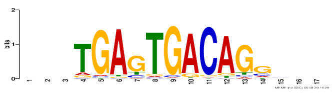

cluster 112

DPRX.MA1480.1

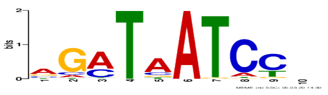

cluster 113

CRX.MA0467.1

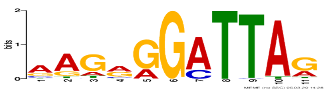

GSC.MA0648.1

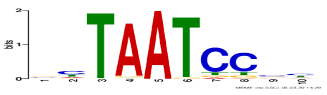

PITX1.MA0682.2

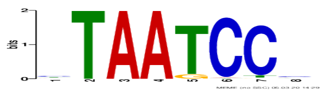

OTX1.MA0711.1

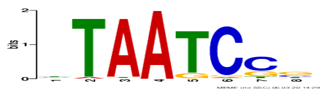

OTX2.MA0712.2

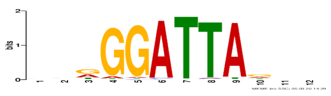

PITX3.MA0714.1

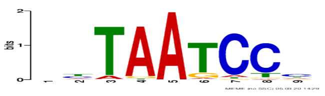

RHOXF1.MA0719.1

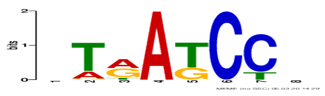

DMBX1.MA0883.1

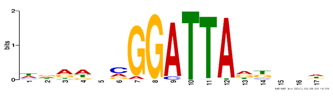

GSC2.MA0891.1

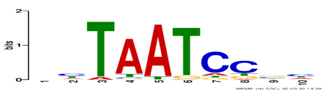

PITX2.MA1547.1

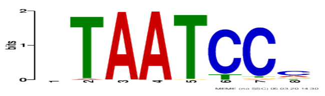

cluster 114

POU5F1_SOX2.MA0142.1

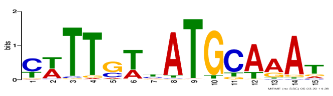

POU2F2.MA0507.1

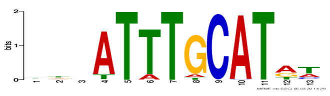

POU2F3.MA0627.2

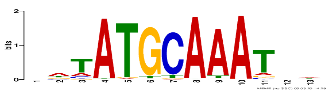

POU1F1.MA0784.1

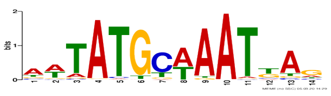

POU2F1.MA0785.1

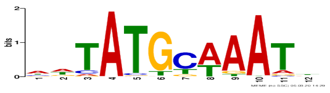

POU3F1.MA0786.1

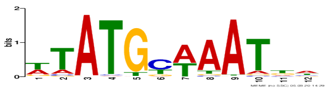

POU3F2.MA0787.1

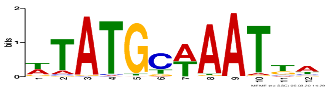

POU3F3.MA0788.1

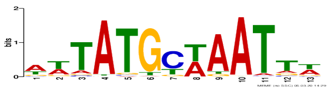

POU3F4.MA0789.1

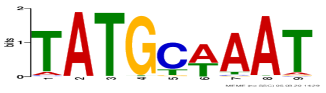

POU5F1B.MA0792.1

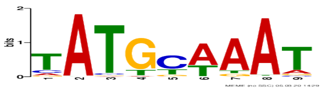

POU5F1.MA1115.1

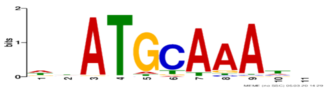

cluster 115

E2F1.MA0024.3

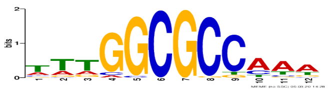

E2F3.MA0469.3

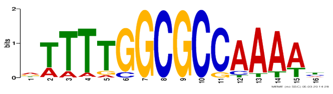

E2F4.MA0470.2

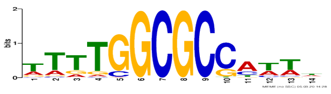

E2F2.MA0864.2

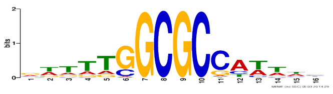

cluster 116

E2F6.MA0471.2

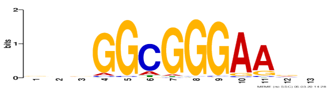

E2F7.MA0758.1

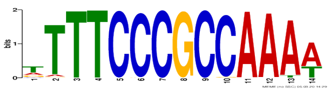

E2F8.MA0865.1

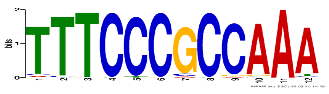

TFDP1.MA1122.1

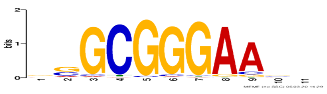

cluster 117

HOXA9.MA0594.2

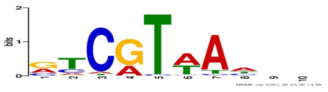

cluster 118

HOXC9.MA0485.2

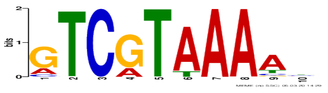

HOXC11.MA0651.1

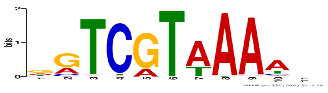

HOXD12.MA0873.1

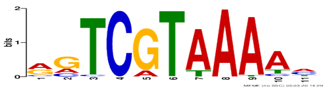

HOXC10.MA0905.1

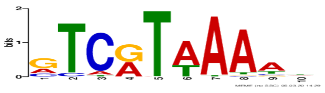

HOXC12.MA0906.1

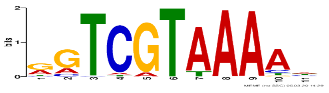

HOXC13.MA0907.1

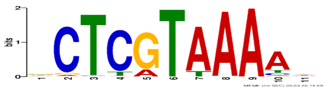

HOXD11.MA0908.1

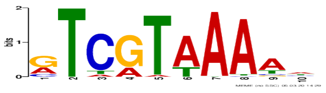

HOXA11.MA0911.1

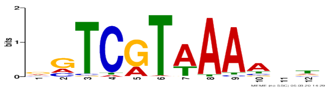

HOXB9.MA1503.1

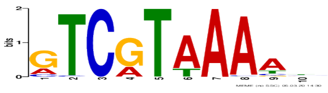

HOXD10.MA1506.1

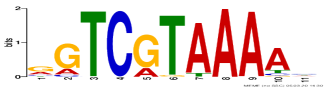

cluster 119

TFAP2A.MA0003.4

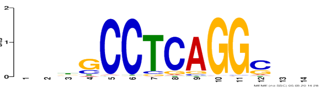

TFAP2B_2.MA0812.1

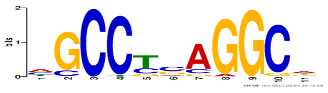

TFAP2C_2.MA0814.2

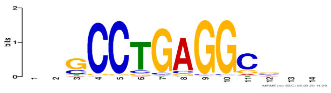

TFAP2E.MA1569.1

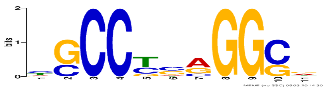

cluster 120

ZNF423.MA0116.1

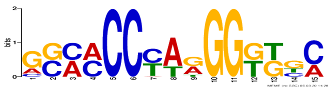

cluster 121

EBF1.MA0154.4

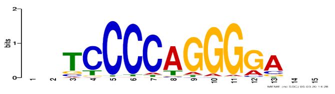

EBF2.MA1604.1

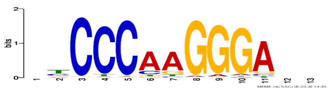

EBF3.MA1637.1

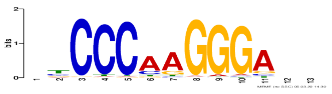

cluster 122

IKZF1.MA1508.1

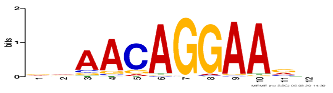

STAT2.MA1623.1

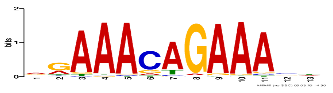

cluster 123

PRDM1.MA0508.3

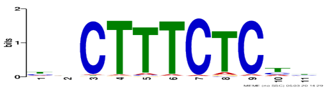

SPIC.MA0687.1

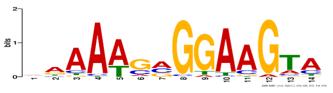

cluster 124

SRY.MA0084.1

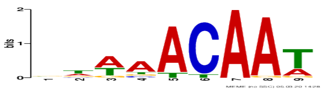

SOX5.MA0087.1

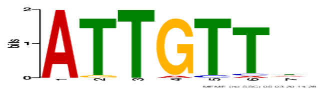

cluster 125

SOX9.MA0077.1

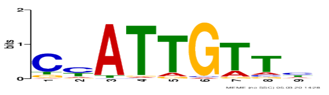

SOX17.MA0078.1

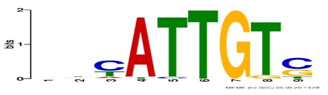

SOX2.MA0143.4

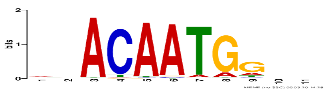

SOX8.MA0868.2

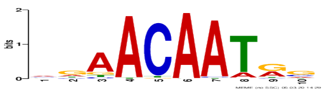

SOX13.MA1120.1

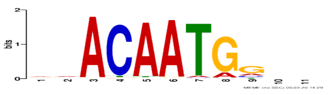

SOX15.MA1152.1

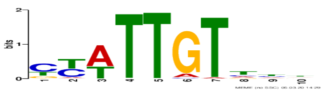

cluster 126

NR1D1.MA1531.1

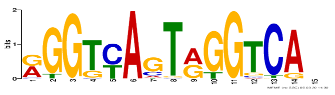

NR1D2.MA1532.1

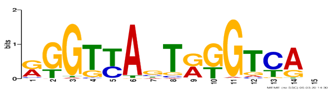

cluster 127

RORA_2.MA0072.1

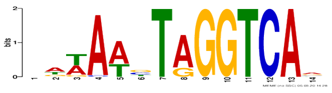

RORB.MA1150.1

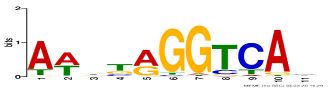

RORC.MA1151.1

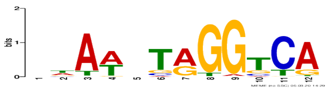

cluster 128

PPARG_RXRA.MA0065.2

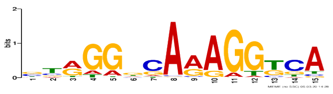

NR1H2_RXRA.MA0115.1

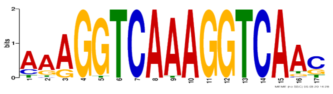

NR2C2.MA0504.1

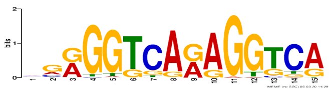

RXRA.MA0512.2

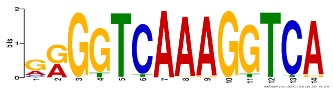

NR2F6.MA0677.1

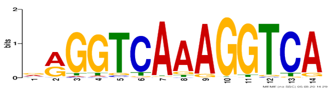

RXRB.MA0855.1

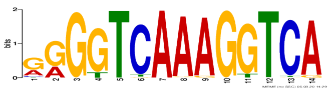

RXRG.MA0856.1

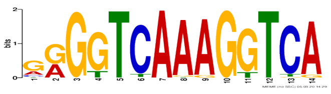

PPARA_RXRA.MA1148.1

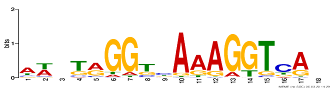

NR2F1_2.MA1537.1

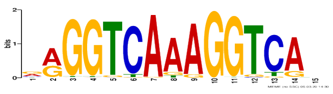

PPARD.MA1550.1

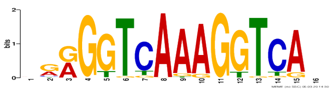

THRB.MA1574.1

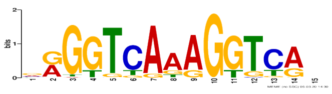

cluster 129

NR1I3.MA1534.1

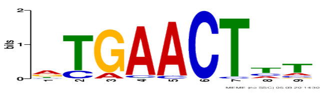

cluster 130

HNF4A.MA0114.4

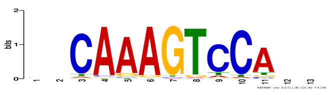

HNF4G.MA0484.2

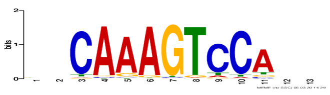

cluster 131

ZNF354C.MA0130.1

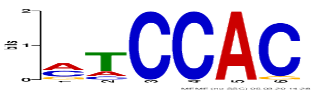

cluster 132

NFYA.MA0060.3

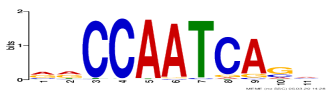

NFYB.MA0502.2

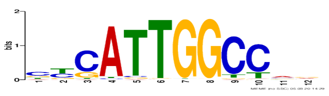

NFYC.MA1644.1

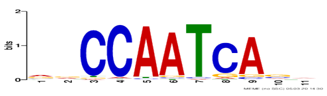

cluster 133

ZNF652.MA1657.1

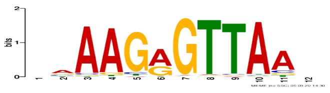

cluster 134

NR2E1.MA0676.1

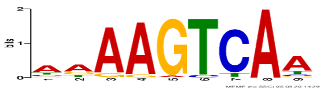

cluster 135

HMBOX1.MA0895.1

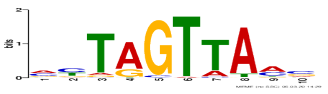

cluster 136

THAP1.MA0597.1

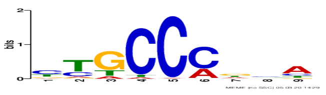

cluster 137

TFAP2C.MA0524.2

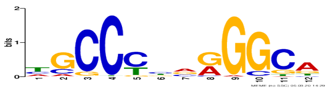

TFAP2A_2.MA0810.1

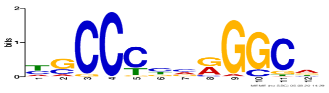

TFAP2B.MA0811.1

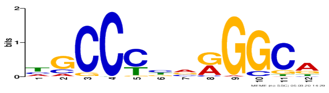

TFAP2B_3.MA0813.1

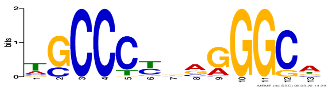

TFAP2C_3.MA0815.1

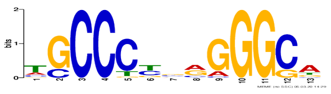

TFAP2A_3.MA0872.1

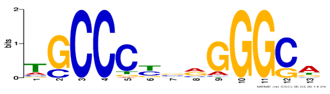

cluster 138

TBXT.MA0009.2

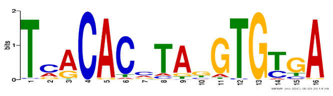

TBX2.MA0688.1

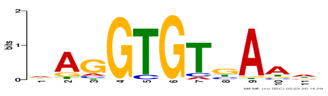

TBX20.MA0689.1

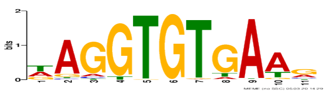

TBX21.MA0690.1

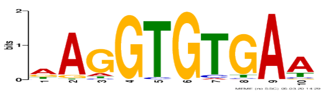

EOMES.MA0800.1

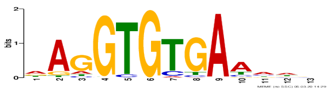

MGA.MA0801.1

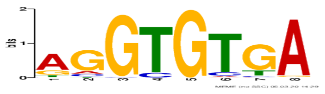

TBR1.MA0802.1

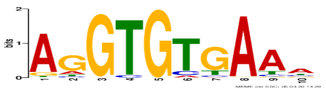

TBX15.MA0803.1

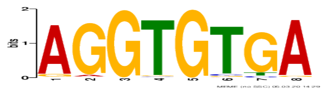

TBX19.MA0804.1

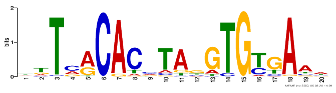

TBX1.MA0805.1

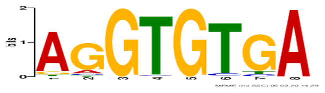

TBX4.MA0806.1

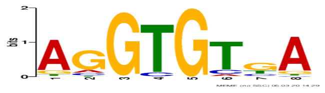

TBX5.MA0807.1

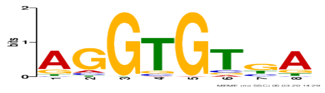

TBX18.MA1565.1

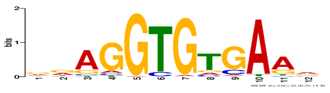

TBX3.MA1566.1

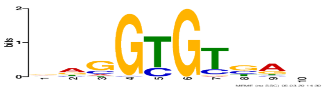

TBX6.MA1567.1

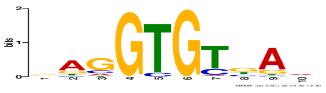

cluster 139

ZBTB33.MA0527.1

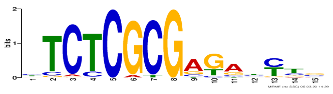

ZBED1.MA0749.1

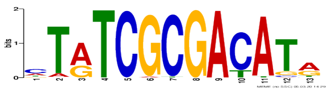

cluster 140

ESRRB.MA0141.3

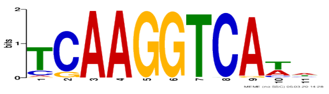

NR5A2.MA0505.1

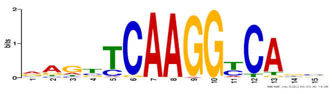

ESRRA.MA0592.3

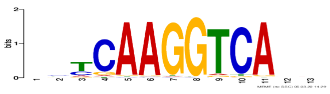

ESRRG.MA0643.1

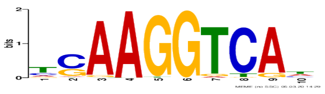

NR2F2.MA1111.1

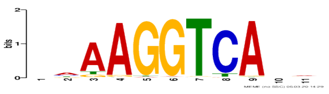

NR4A1.MA1112.2

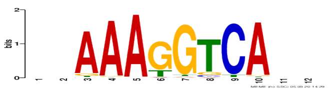

NR5A1.MA1540.1

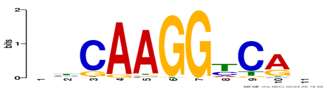

NR6A1.MA1541.1

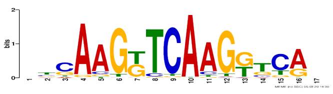

cluster 141

NR1H3_RXRA.MA0494.1

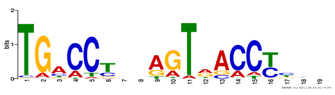

RARG_2.MA0860.1

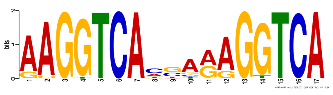

THRB_3.MA1576.1

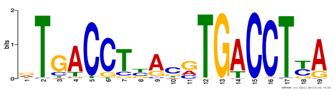

cluster 142

RARA_RXRA.MA0159.1

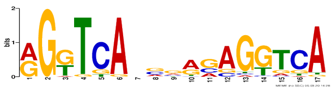

RARA_2.MA0730.1

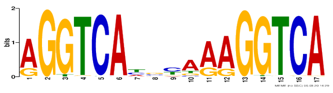

RARB_2.MA0858.1

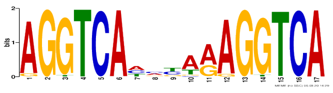

RARA_RXRG.MA1149.1

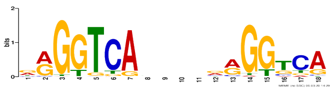

cluster 143

NR2F1.MA0017.2

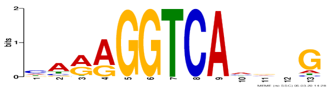

RORA.MA0071.1

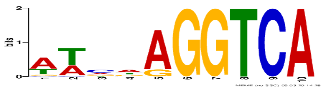

NR4A2.MA0160.1

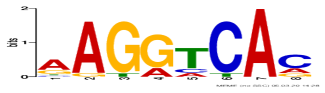

NR2C1.MA1535.1

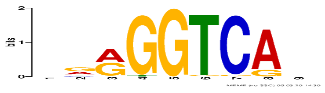

NR2C2_2.MA1536.1

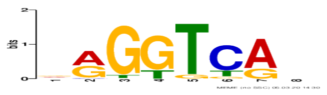

THRB_2.MA1575.1

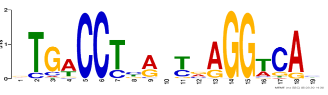

cluster 144

KLF4.MA0039.4

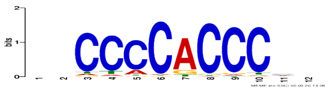

ZNF263.MA0528.2

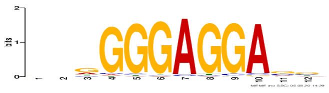

KLF5.MA0599.1

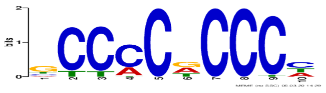

ZNF740.MA0753.2

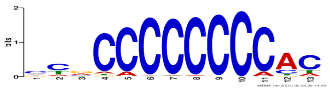

KLF15.MA1513.1 | MAZ.MA1522.1 | VEZF1.MA1578.1 | ZNF148.MA1653.1

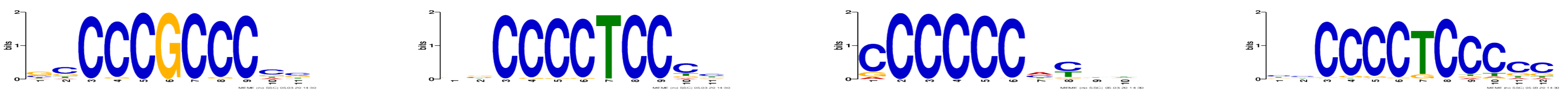

cluster 145

FOXQ1.MA0040.1 | FOXD3.MA0041.1 | FOXE1.MA1487.1

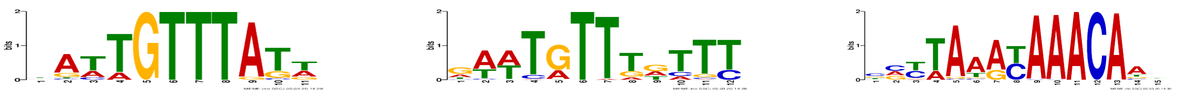

cluster 146

FOXF2.MA0030.1 | FOXD1.MA0031.1 | FOXC1.MA0032.2 | FOXL1.MA0033.2

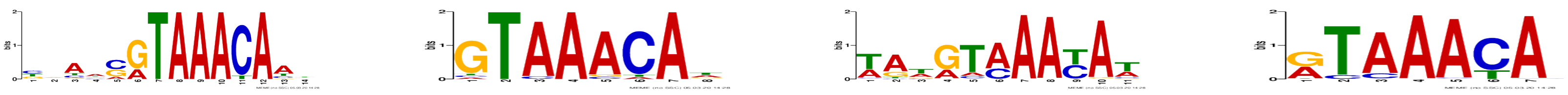

FOXI1.MA0042.2 | FOXA2.MA0047.3 | FOXA1.MA0148.4 | FOXO3.MA0157.2

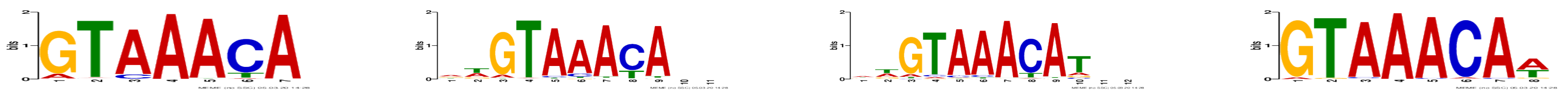

FOXO1.MA0480.1 | FOXP1.MA0481.3 | FOXP2.MA0593.1 | FOXG1.MA0613.1

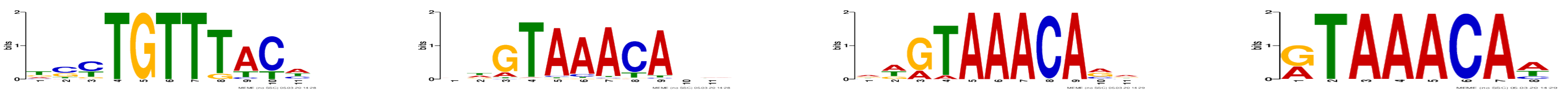

FOXJ2.MA0614.1 | FOXB1.MA0845.1 | FOXC2.MA0846.1 | FOXD2.MA0847.2

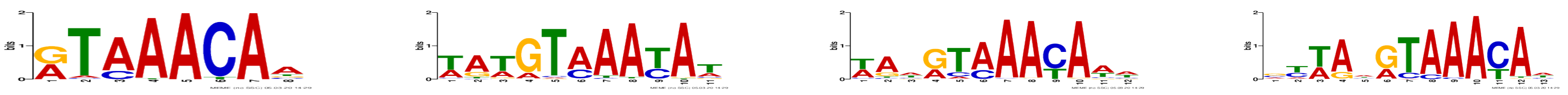

FOXO4.MA0848.1 | FOXO6.MA0849.1 | FOXP3.MA0850.1 | FOXJ3.MA0851.1

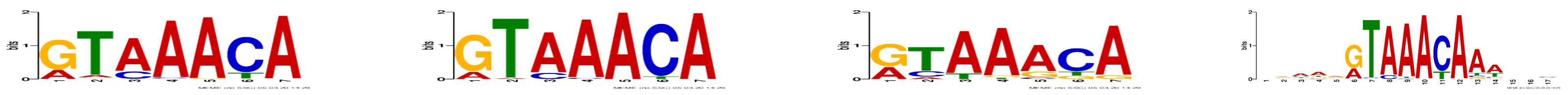

FOXK1.MA0852.2 | FOXK2.MA1103.2 | FOXN3.MA1489.1 | FOXF1.MA1606.1

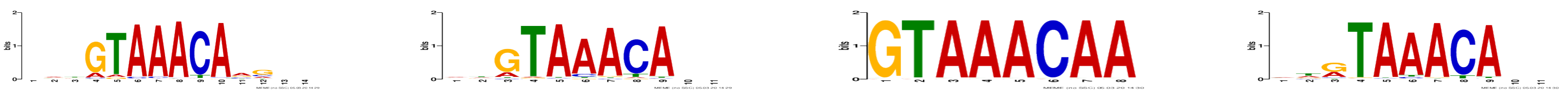

FOXL2.MA1607.1 | FOXA3.MA1683.1

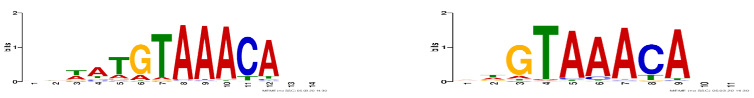

cluster 147

SP1.MA0079.4 | KLF1.MA0493.1 | SP2.MA0516.2 | KLF13.MA0657.1

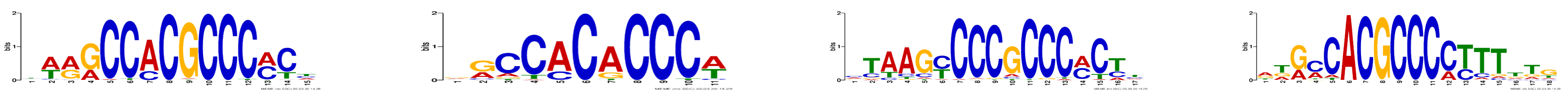

SP4.MA0685.1 | KLF14.MA0740.1 | KLF16.MA0741.1 | KLF12.MA0742.1

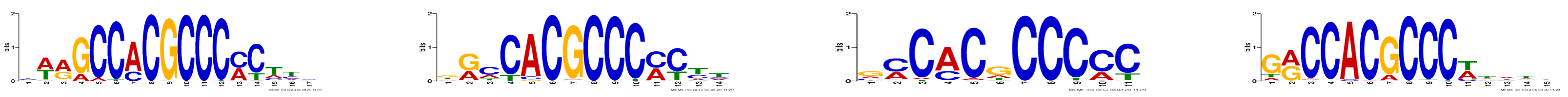

SP3.MA0746.2 | SP8.MA0747.1 | KLF9.MA1107.2 | KLF10.MA1511.1

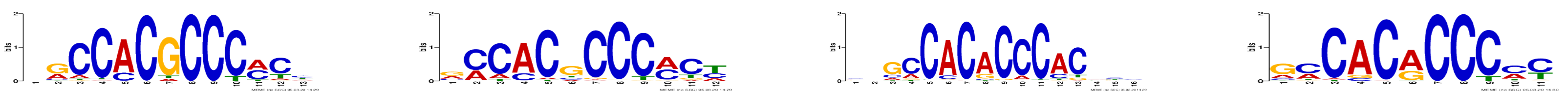

KLF11.MA1512.1

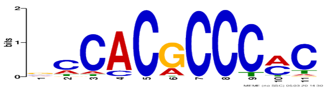

KLF2.MA1515.1

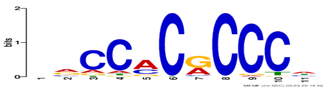

KLF3.MA1516.1

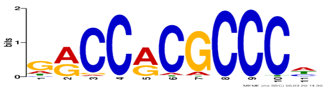

KLF6.MA1517.1

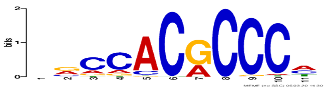

SP9.MA1564.1

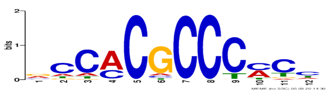

cluster 148

EGR1.MA0162.4

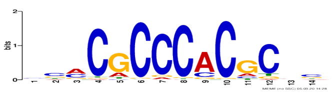

EGR2.MA0472.2

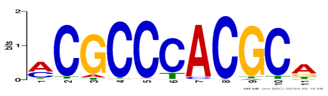

EGR3.MA0732.1

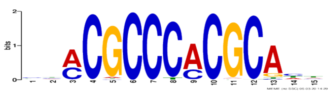

EGR4.MA0733.1

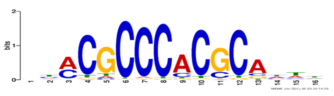

cluster 149

AHR_ARNT.MA0006.1

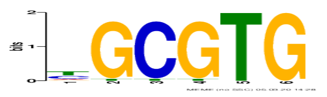

KLF17.MA1514.1

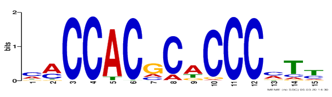

cluster 150

RXRA_VDR.MA0074.1

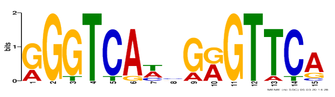

VDR.MA0693.2

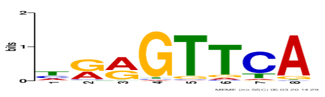

NR1I2.MA1533.1

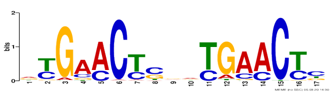

cluster 151

RARB_3.MA1552.1

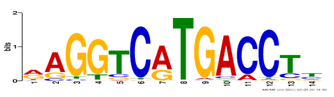

RARG_3.MA1553.1

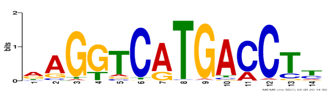

RXRB_2.MA1555.1

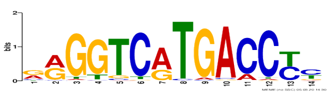

RXRG_2.MA1556.1

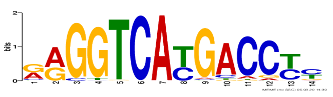

cluster 152

NR1H4.MA1110.1

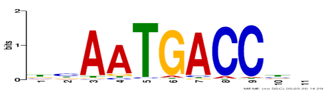

NR1H4_RXRA.MA1146.1

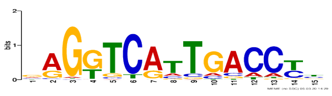

NR4A2_RXRA.MA1147.1

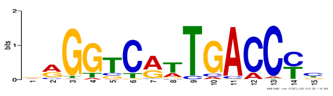

NR2F1_3.MA1538.1

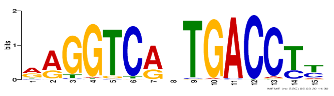

NR2F6_3.MA1539.1

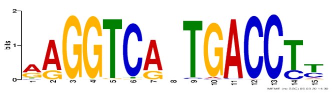

cluster 153

ELK1.MA0028.2

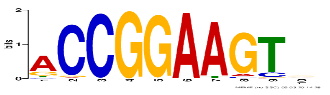

ETS1.MA0098.3

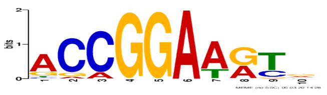

FEV.MA0156.2

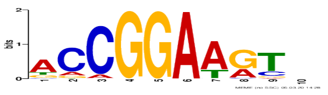

ERG.MA0474.2

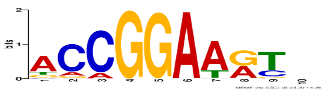

FLI1.MA0475.2

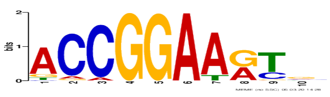

ELF4.MA0641.1

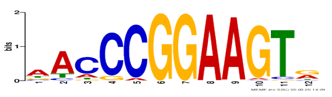

SPDEF.MA0686.1

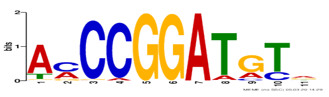

ELK3.MA0759.1

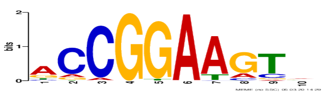

ERF.MA0760.1

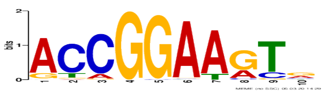

ETV2.MA0762.1

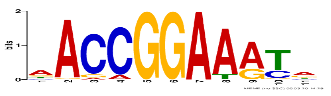

ETV3.MA0763.1

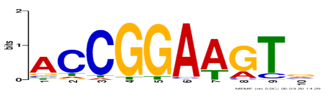

ELF2.MA1483.1

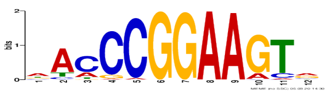

ETS2.MA1484.1

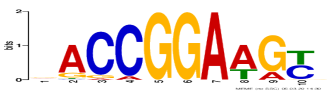

cluster 154

MAFB.MA0117.2

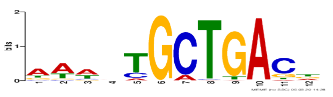

MAFF.MA0495.3

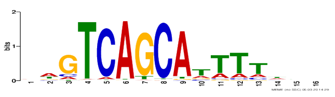

MAFG.MA0659.2

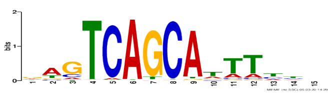

NRL.MA0842.2

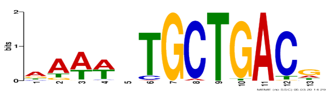

cluster 155

NFE2L1.MA0089.2

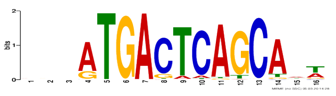

NFE2L2.MA0150.2

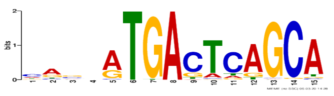

MAFK.MA0496.3

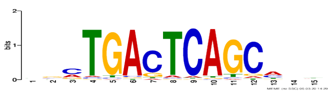

MAF_NFE2.MA0501.1

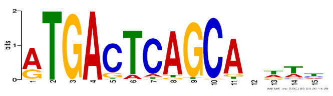

BACH1_MAFK.MA0591.1

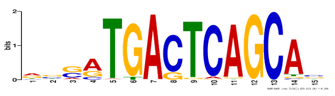

MAF.MA1520.1

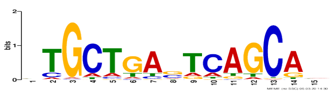

MAFA.MA1521.1

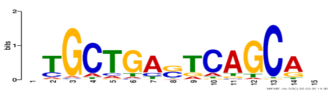

BACH1.MA1633.1

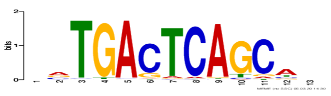

cluster 156

ZKSCAN5.MA1652.1

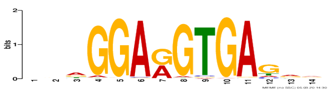

cluster 157

GABPA.MA0062.3

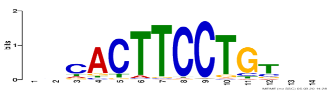

ELK4.MA0076.2

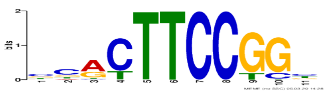

SPI1.MA0080.5

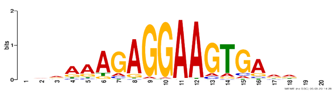

SPIB.MA0081.2

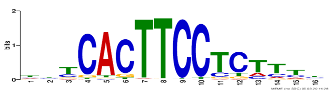

ELF5.MA0136.2

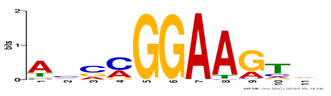

ELF1.MA0473.3

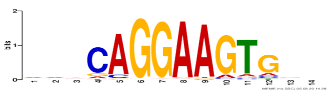

EHF.MA0598.3

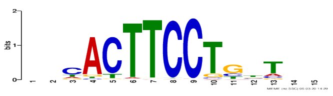

ELF3.MA0640.2

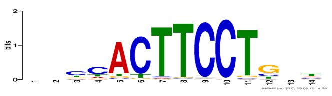

ETV6.MA0645.1

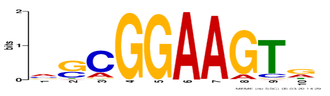

ZBTB7A.MA0750.2

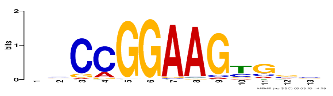

ETV1.MA0761.2

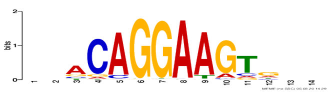

ETV4.MA0764.2

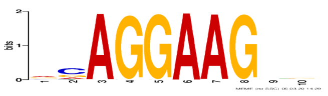

ETV5.MA0765.2

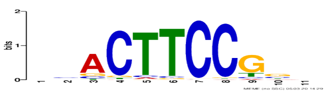

cluster 158

NR2F6_2.MA0728.1

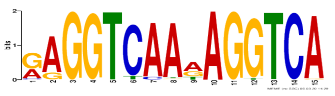

RARA.MA0729.1

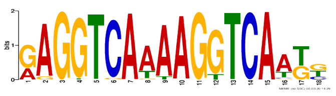

RARB.MA0857.1

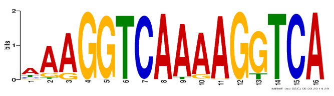

RARG.MA0859.1

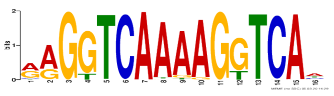

HNF4A_2.MA1494.1

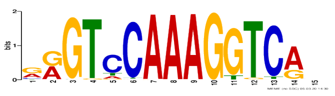

cluster 159

ZNF274.MA1592.1

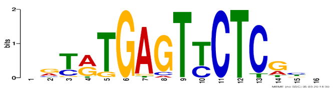

cluster 160

FOS_JUN.MA0099.3

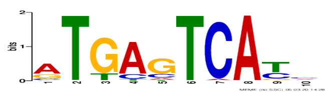

BATF_JUN.MA0462.2

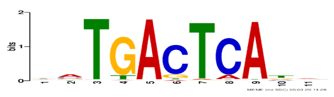

FOS.MA0476.1

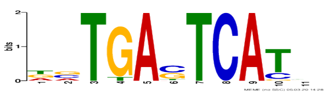

FOSL1.MA0477.2

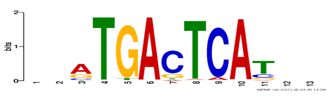

FOSL2.MA0478.1

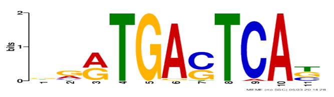

JUN_2.MA0489.1

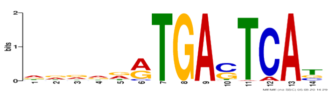

JUNB.MA0490.2

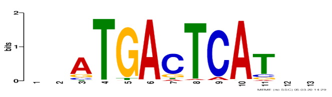

JUND.MA0491.2

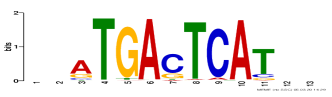

JDP2.MA0655.1

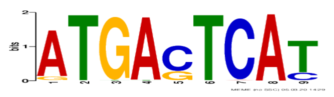

BATF3.MA0835.2

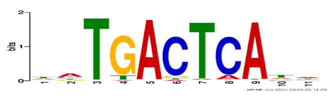

NFE2.MA0841.1

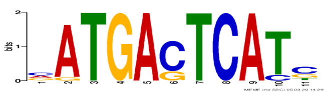

BACH2.MA1101.2

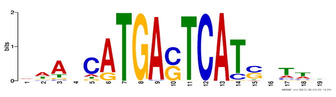

FOSL1_JUN.MA1128.1

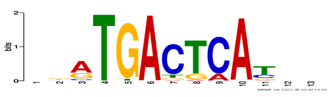

FOSL2_JUN.MA1130.1

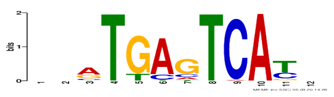

JUN_JUNB.MA1132.1

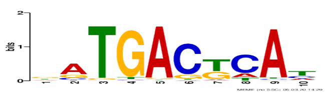

FOS_JUNB.MA1134.1

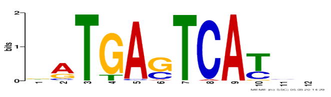

FOSB_JUNB.MA1135.1

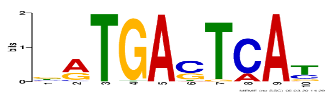

FOSL1_JUNB.MA1137.1

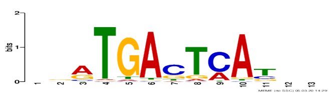

FOSL2_JUNB.MA1138.1

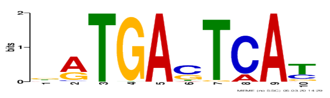

FOS_JUND.MA1141.1

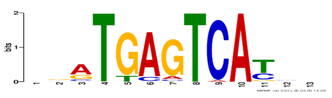

FOSL1_JUND.MA1142.1

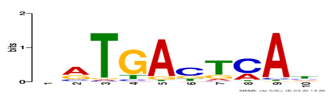

FOSL2_JUND.MA1144.1

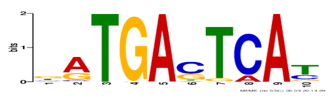

SMAD2_SMAD3.MA1622.1

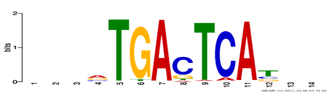

BATF.MA1634.1

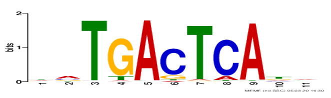

cluster 161

GMEB1.MA0615.1

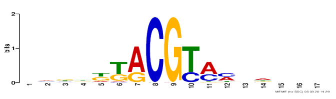

GMEB2.MA0862.1

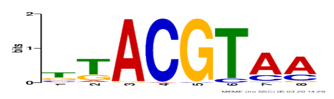

cluster 162

HNF1A.MA0046.2

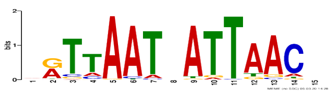

HNF1B.MA0153.2

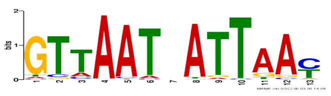

POU4F2.MA0683.1

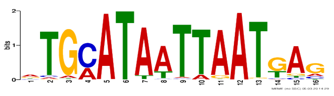

POU4F1.MA0790.1

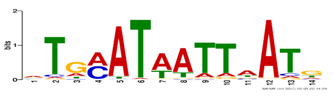

POU4F3.MA0791.1

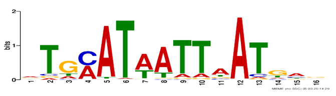

cluster 163

ZNF24.MA1124.1

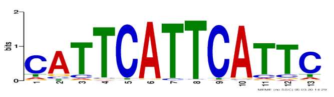

cluster 164

PBX1.MA0070.1

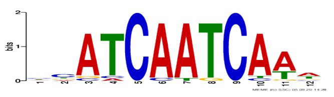

PBX2.MA1113.2

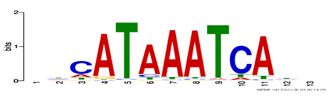

MEIS1_2.MA1639.1

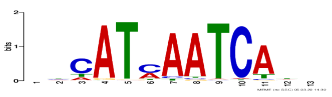

MEIS2_2.MA1640.1

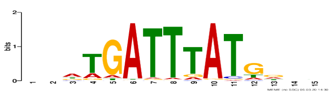

cluster 165

PPARG.MA0066.1

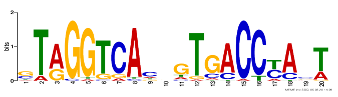

ESR1.MA0112.3

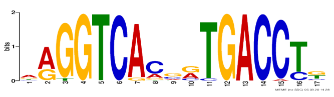

ESR2.MA0258.2

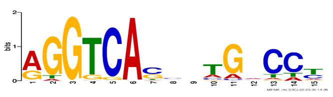

cluster 166

PAX2.MA0067.1

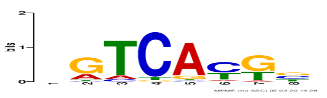

cluster 167

PAX5.MA0014.3

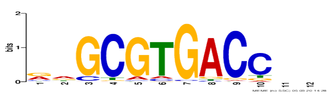

PAX3_2.MA1546.1

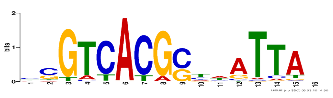

cluster 168

BARHL2.MA0635.1

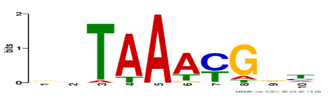

BARHL1.MA0877.2

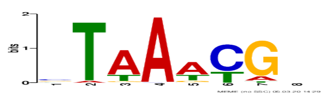

cluster 169

MSX1.MA0666.1

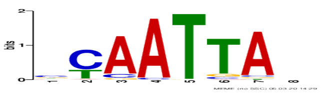

MSX2.MA0708.1

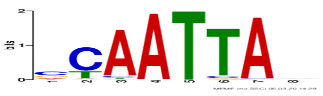

MSX3.MA0709.1

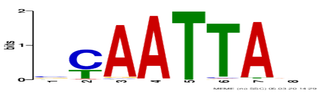

VENTX.MA0724.1

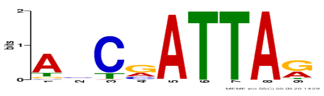

BARX1.MA0875.1

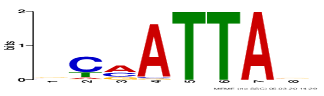

BSX.MA0876.1

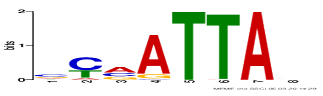

DLX3.MA0880.1

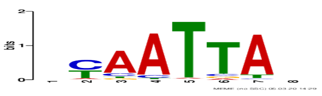

DLX4.MA0881.1

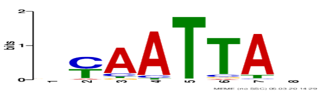

DLX6.MA0882.1

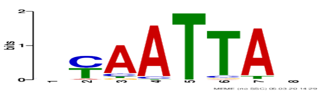

DLX2.MA0885.1

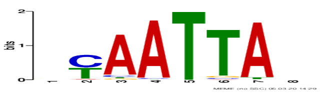

BARX2.MA1471.1

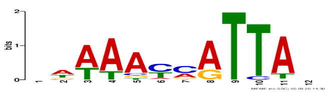

DLX5.MA1476.1

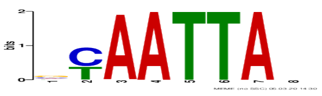

LHX1.MA1518.1

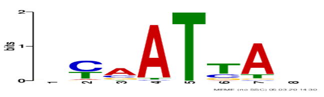

cluster 170

ATF1.MA0604.1

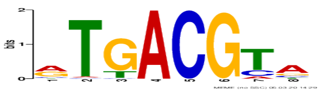

FOS_JUN_2.MA1126.1

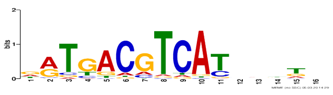

FOSL1_JUN_2.MA1129.1

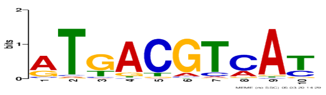

JUN_JUNB_2.MA1133.1

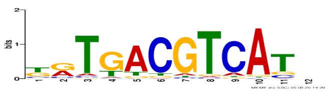

FOSB_JUNB_2.MA1136.1

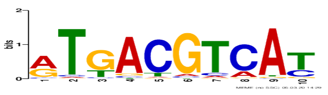

FOSL1_JUND_2.MA1143.1

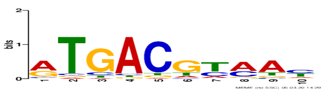

cluster 171

CREB1.MA0018.4

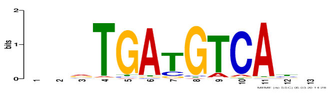

JUN.MA0488.1

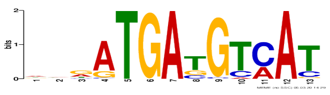

JUND_2.MA0492.1

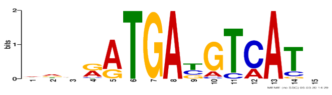

ATF3.MA0605.2

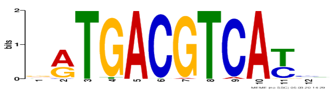

CREM.MA0609.2

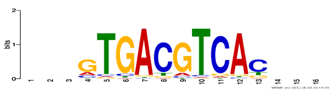

JDP2_2.MA0656.1

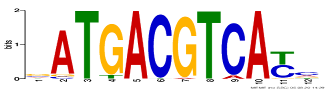

ATF7.MA0834.1

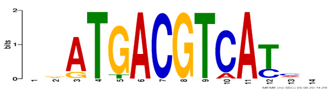

CREB5.MA0840.1

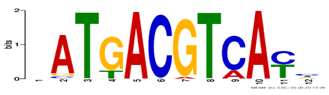

FOSB_JUN.MA1127.1

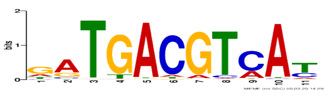

FOSL2_JUN_2.MA1131.1

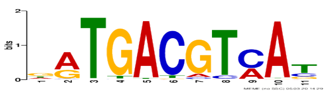

FOSL2_JUNB_2.MA1139.1

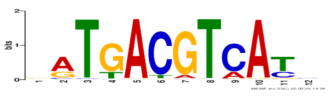

JUNB_2.MA1140.2

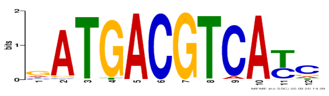

FOSL2_JUND_2.MA1145.1

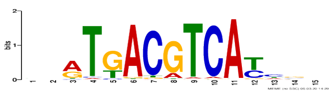

BACH2_2.MA1470.1

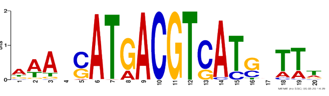

CREB3L4_2.MA1475.1

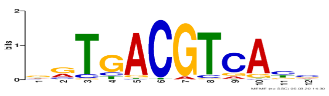

ATF2.MA1632.1

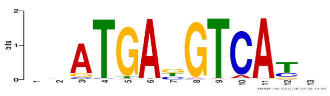

cluster 172

ARNT_HIF1A.MA0259.1

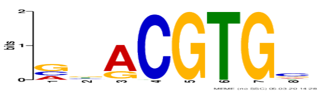

CREB3L2.MA0608.1

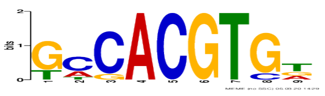

HES2.MA0616.2

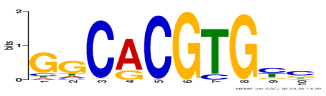

MLXIP.MA0622.1

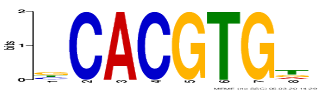

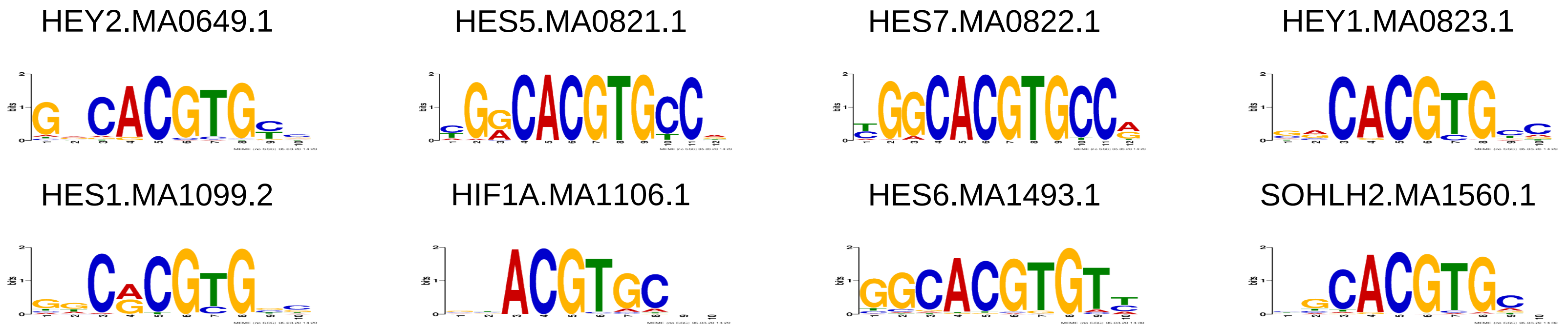

cluster 173

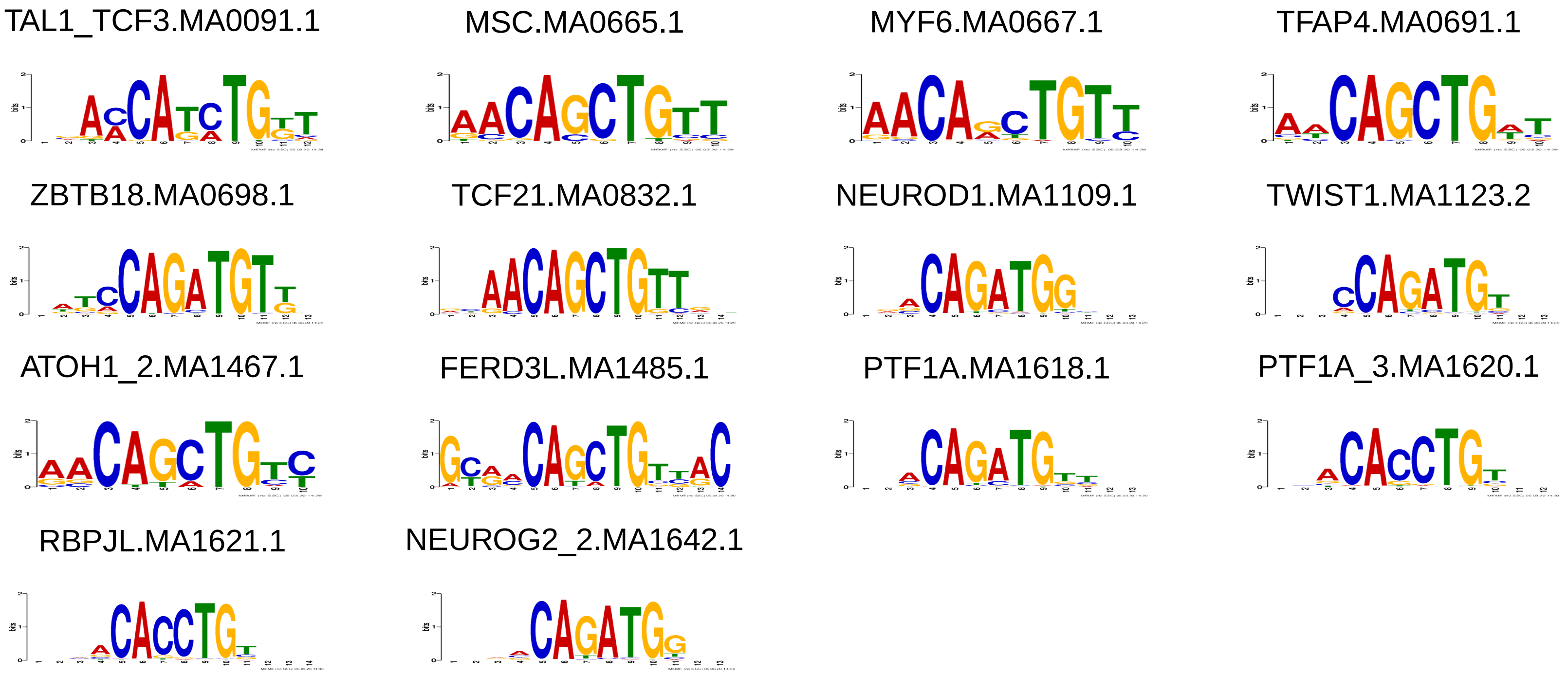

cluster 174

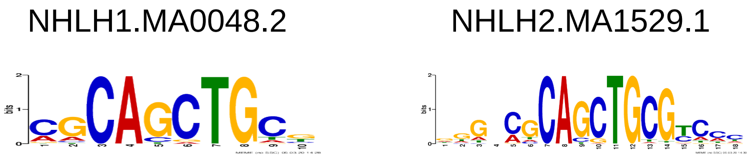

cluster 175

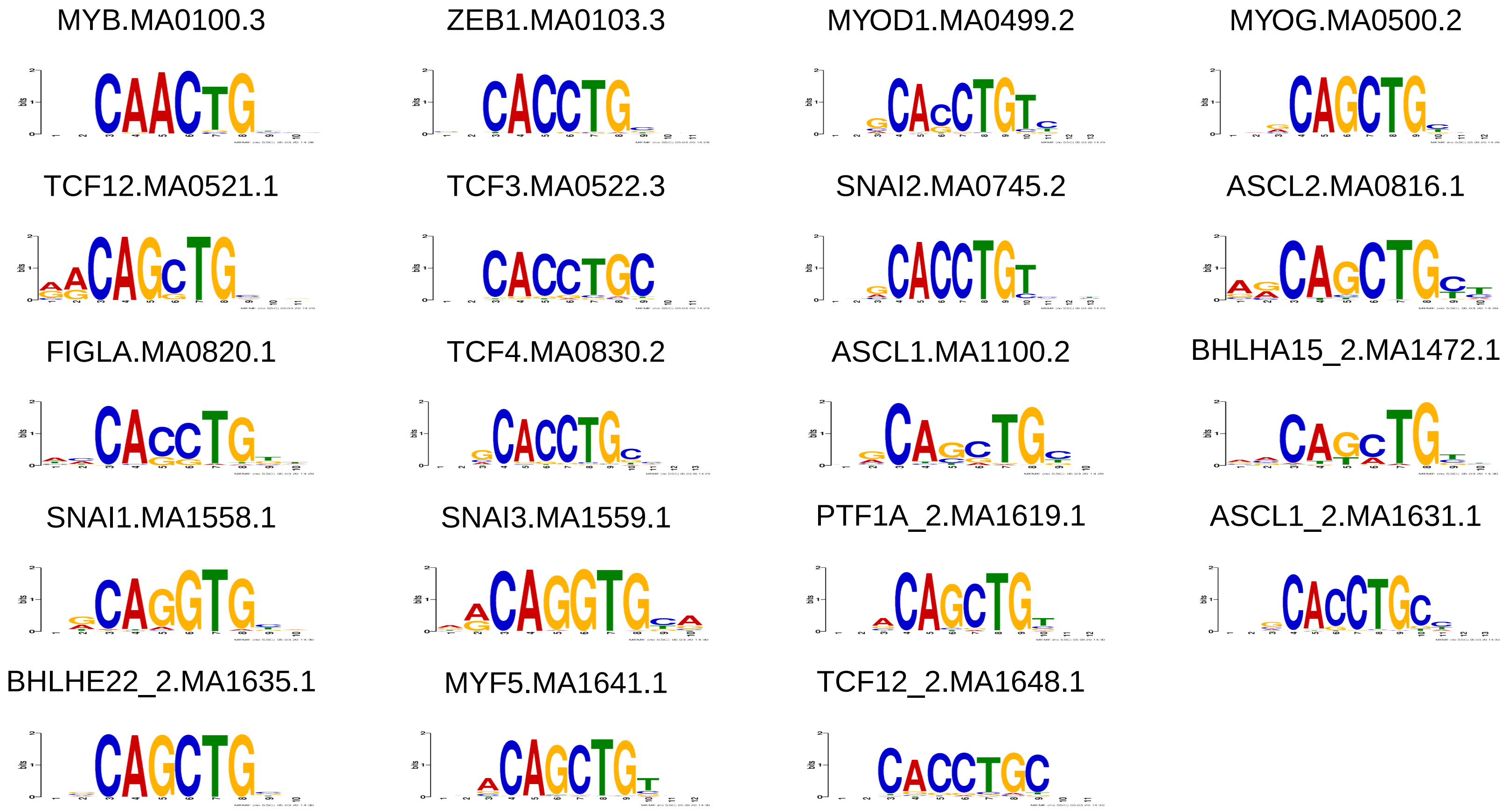

cluster 176

CREB3.MA0638.1

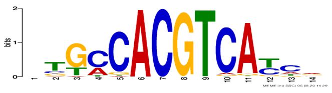

CREB3L1.MA0839.1

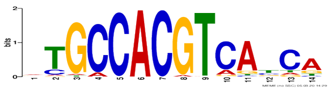

XBP1.MA0844.1

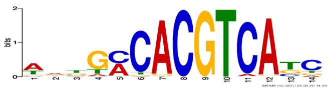

ATF6.MA1466.1

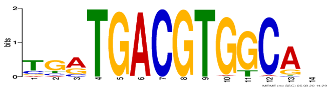

CREB3L4.MA1474.1

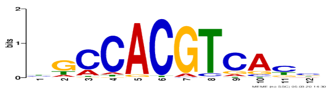

cluster 177

ARNT.MA0004.1

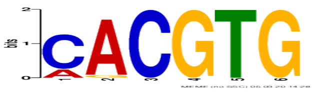

MAX.MA0058.3

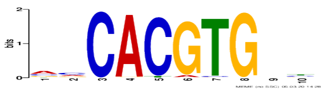

MAX_MYC.MA0059.1

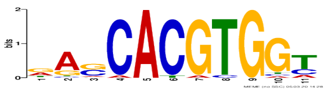

USF1.MA0093.3

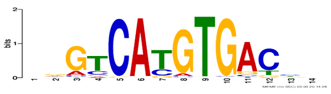

MYCN.MA0104.4

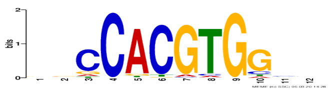

MYC.MA0147.3

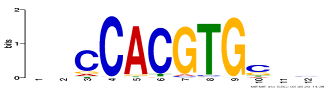

BHLHE40.MA0464.2

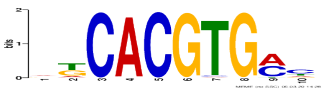

USF2.MA0526.3

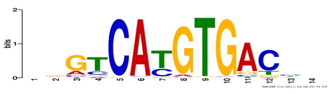

ARNTL.MA0603.1

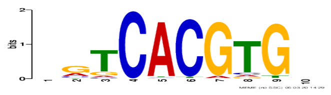

MITF.MA0620.3

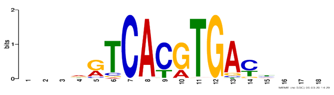

NPAS2.MA0626.1

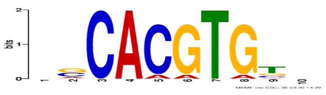

BHLHE41.MA0636.1

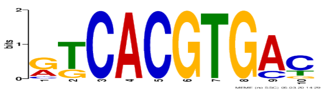

MLX.MA0663.1

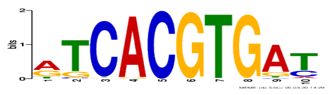

MLXIPL.MA0664.1

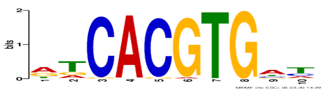

TFEB.MA0692.1

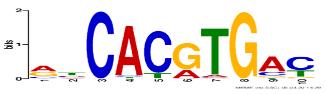

CLOCK.MA0819.1

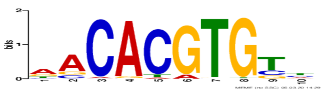

MNT.MA0825.1

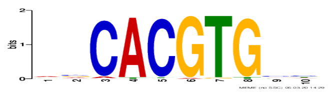

SREBF2_2.MA0828.1

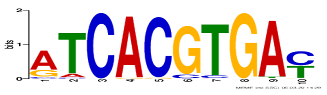

SREBF1_2.MA0829.2

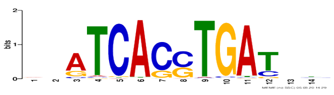

TFE3.MA0831.2

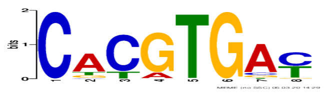

TFEC.MA0871.2

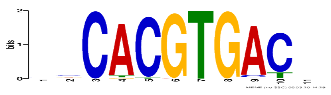

ARNT2.MA1464.1

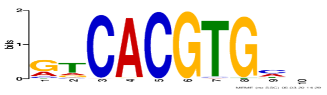

cluster 178

DUX4.MA0468.1

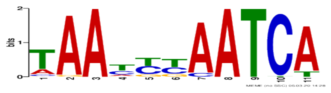

PHOX2B.MA0681.2

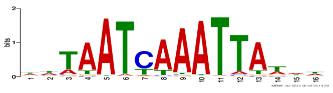

PHOX2A.MA0713.1

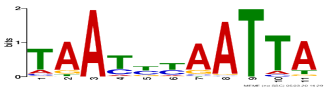

PROP1.MA0715.1

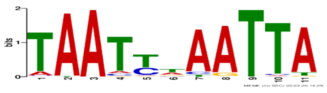

DUXA.MA0884.1

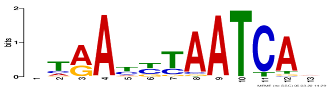

cluster 179

LHX3.MA0135.1

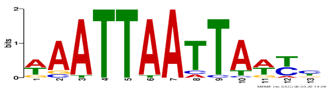

ARID3B.MA0601.1

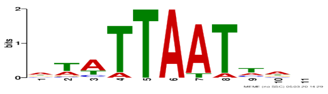

MIX_A.MA0621.1

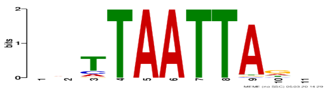

LMX1A.MA0702.2

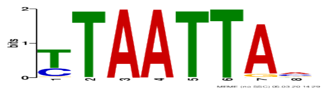

LMX1B.MA0703.2 ALX4.MA0853.1 ALX1.MA0854.1 ARX.MA0874.1

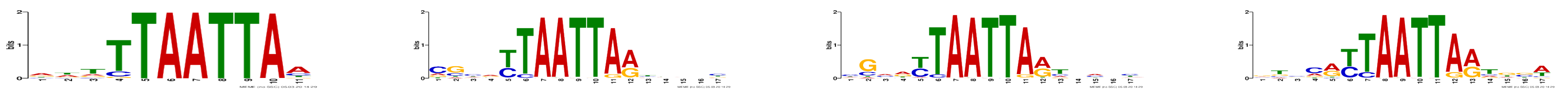

ARGFX.MA1463.1

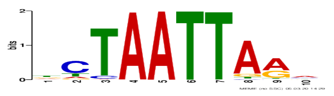

cluster 180

EN1.MA0027.2 PAX4.MA0068.2 PRRX2.MA0075.3 NOBOX.MA0125.1

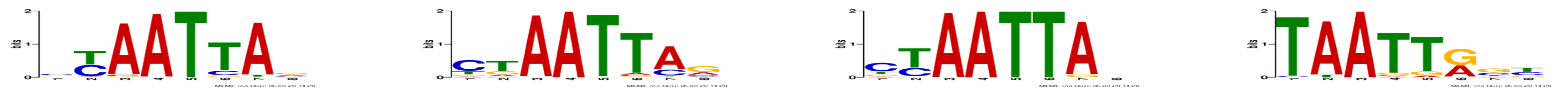

PDX1.MA0132.2 HOXA5.MA0158.2 EMX1.MA0612.2 LBX1.MA0618.1

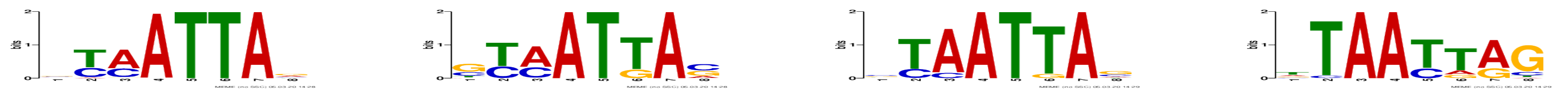

POU6F1.MA0628.1 SHOX.MA0630.1 ALX3.MA0634.1 EN2.MA0642.1

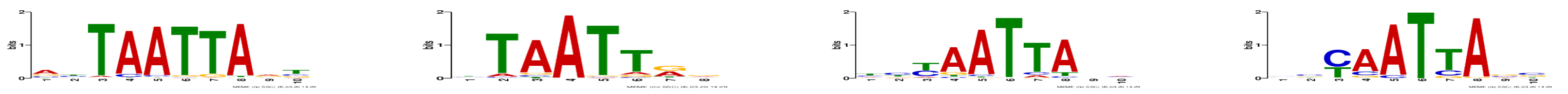

ESX1.MA0644.1 ISX.MA0654.1 LHX6.MA0658.1 MEOX1.MA0661.1

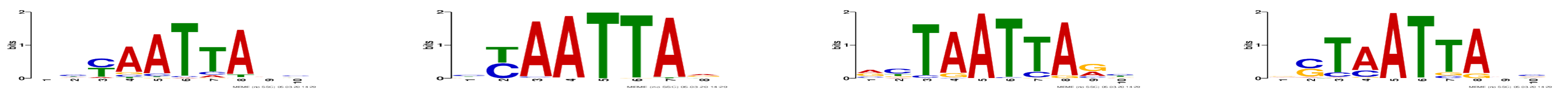

MIXL1.MA0662.1 NKX6_1.MA0674.1 NKX6_2.MA0675.1 LBX2.MA0699.1

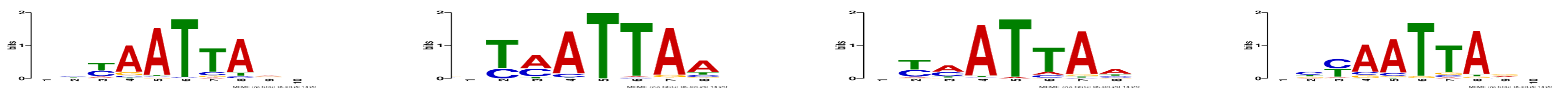

LHX9.MA0701.2 LHX4.MA0704.1 LHX8.MA0705.1 MEOX2.MA0706.1

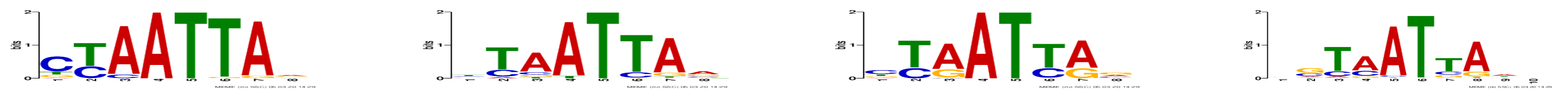

MNX1.MA0707.1 NOTO.MA0710.1 PRRX1.MA0716.1 RAX2.MA0717.1

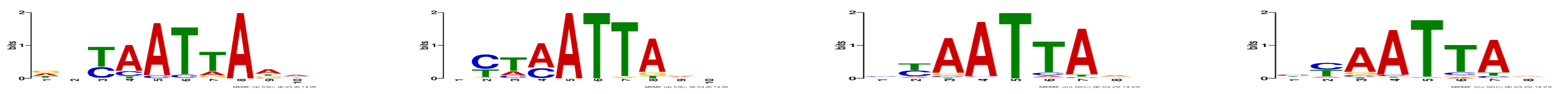

RAX.MA0718.1 SHOX2.MA0720.1 UNCX.MA0721.1 VAX1.MA0722.1

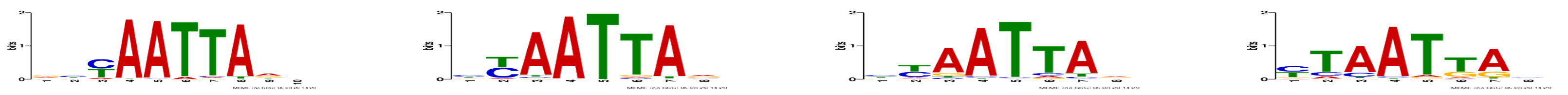

VAX2.MA0723.1 VSX1.MA0725.1 VSX2.MA0726.1 POU6F2.MA0793.1

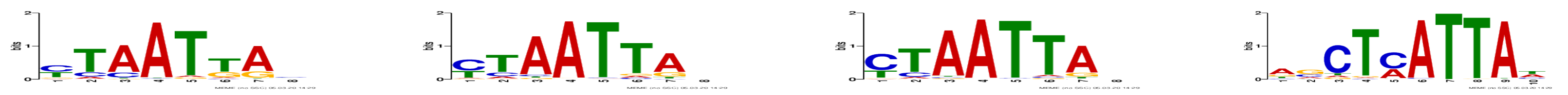

DLX1.MA0879.1 EMX2.MA0886.1 EVX1.MA0887.1 EVX2.MA0888.1

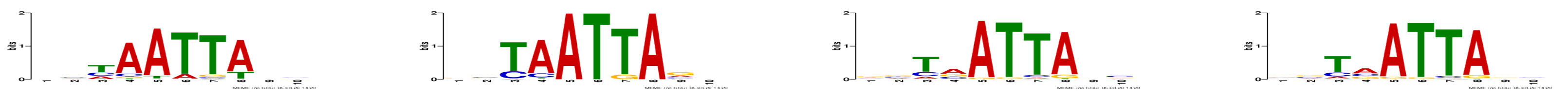

GBX1.MA0889.1 GBX2.MA0890.1 GSX1.MA0892.1 GSX2.MA0893.2

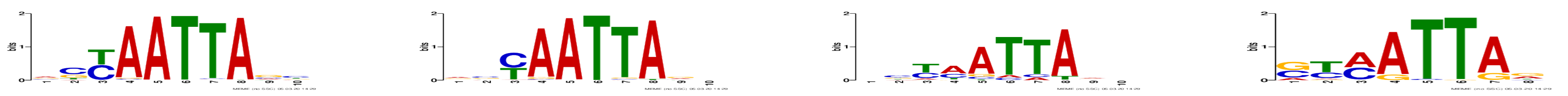

HESX1.MA0894.1 HOXA2.MA0900.2 HOXB2.MA0902.2 HOXB3.MA0903.1

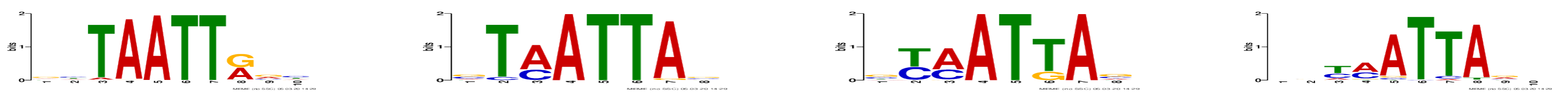

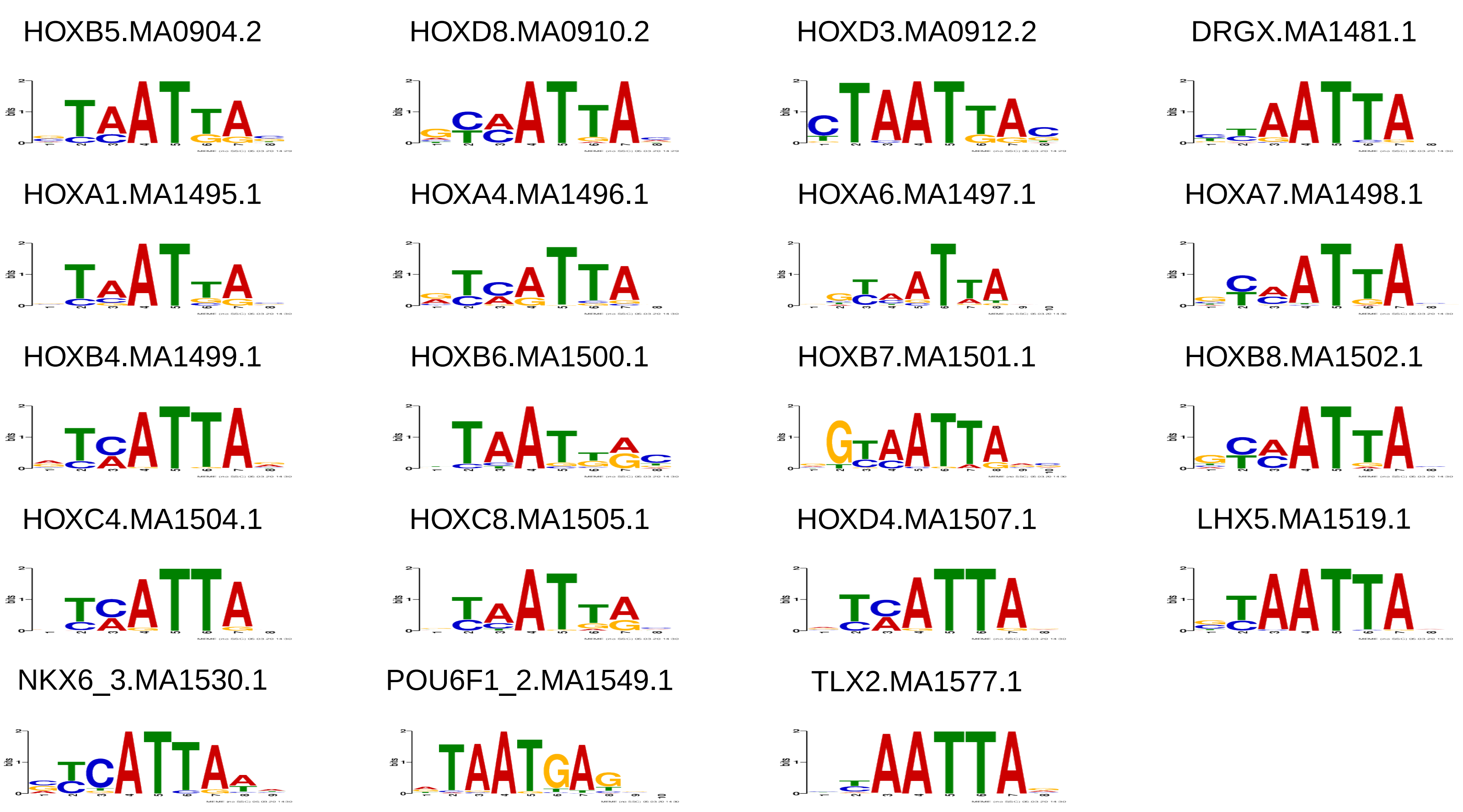
HOXB5.MA0904.2
HOXD8.MA0910.2
HOXD3.MA0912.2
DRGX.MA1481.1
HOXA1.MA1495.1
HOXA4.MA1496.1
HOXA6.MA1497.1
HOXA7.MA1498.1
HOXB4.MA1499.1
HOXB6.MA1500.1
HOXB7.MA1501.1
HOXB8.MA1502.1
HOXC4.MA1504.1
HOXC8.MA1505.1
HOXD4.MA1507.1
LHX5.MA1519.1
NKX6_3.MA1530.1
POU6F1_2.MA1549.1
TLX2.MA1577.1
