# Supplementary material for: TFmotifView: a webserver for the visualization of transcription factor motifs in genomic regions
Source: Nucleic Acids Res. 2020 Apr 23;48(W1):W208–17. doi: 10.1093/nar/gkaa252 (PMC7319436; doi:10.1093/nar/gkaa252)
Supplement: gkaa252_Supplemental_Files [file gkaa252_supplemental_files.zip › Supplementary_Figure1_motif_information_content.pdf]

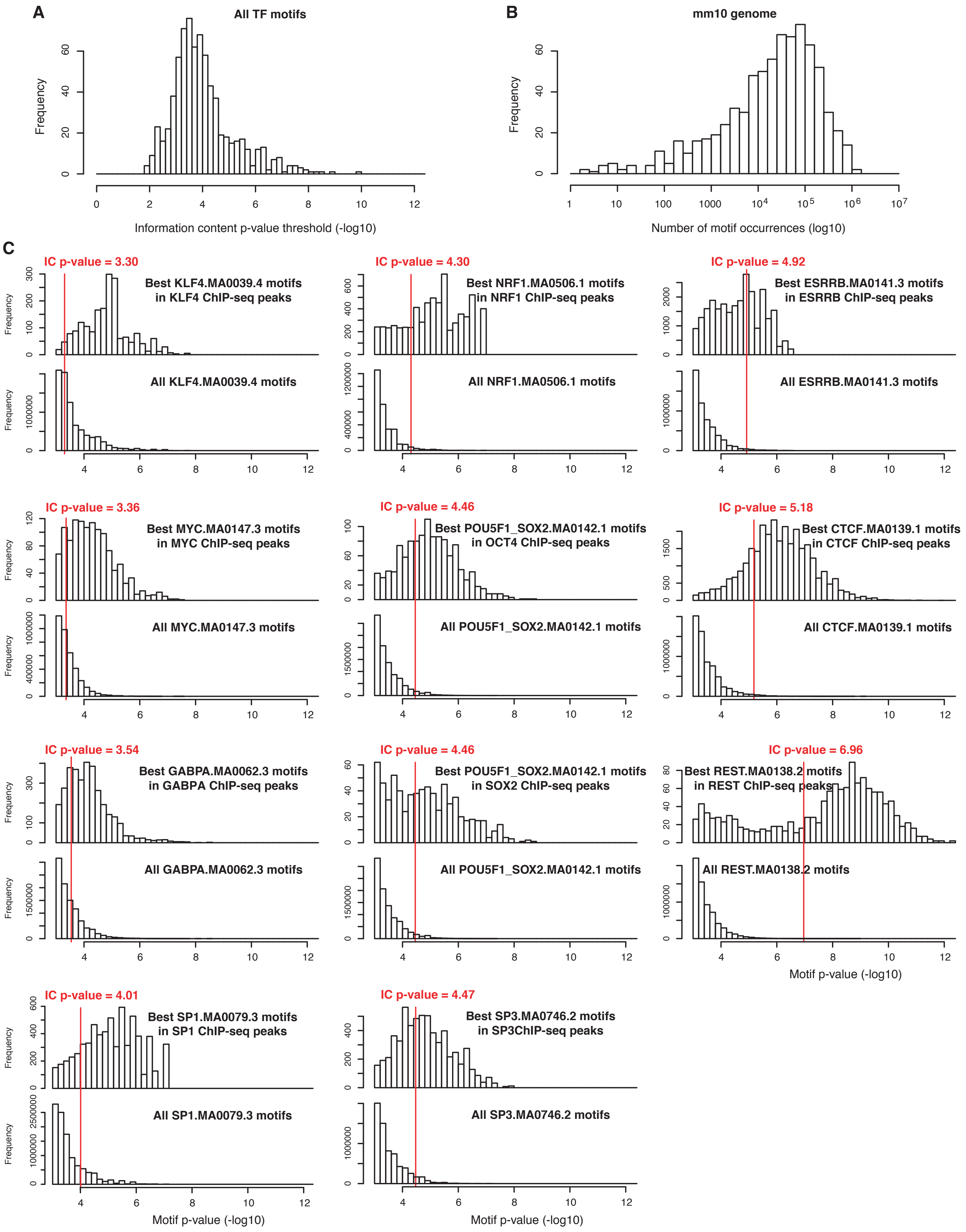

Supplementary figure 1 - Motif information content based p-value thresholds
A. Distribution of all motif's information content used as p-value thresholds.
B. Distribution of the number of TF motif occurrences using information content thresholds in the mm10 genome.
C. Illustration of the choice of motif's information content as dynamic p-value threshold. For TFs expressed in mouse embryonic stem cells, the distribution of p-values of the best motif occurrences present in corresponding ChIP-seq peaks is shifted towards better p-values compared to all motif occurrences found in the mouse genome. The information content based p-value thresholds (red lines), computed automatically from the motif PWMs, separates well the distribution of motif p-values in peaks versus all. Rather than using the same lenient p-value threshold for all motifs, the use of this dynamic motif information content based p-value threshold enables to minimize the number of false positive predictions.
